# Free three-dimensional carborane carbanions†

**DOI:** 10.1039/d1sc02252k

**Published:** 2021-06-30

**Authors:** H. D. A. Chathumal Jayaweera, Md. Mamdudur Rahman, Perry J. Pellechia, Mark D. Smith, Dmitry V. Peryshkov

**Affiliations:** Department of Chemistry and Biochemistry, University of South Carolina 631 Sumter St. Columbia South Carolina 29208 USA peryshkov@sc.edu

## Abstract

Carbon atom functionalization *via* generation of carbanions is the cornerstone of carborane chemistry. In this work, we report the synthesis and structural characterization of free *ortho*-carboranyl [C_2_B_10_H_11_]^−^, a three-dimensional inorganic analog of the elusive phenyl anion that features a “naked” carbanion center. The first example of a stable, discrete C(H)-deprotonated carborane anion was isolated as a completely separated ion pair with a crown ether-encapsulated potassium cation. An analogous approach led to the isolation and structural characterization of a doubly deprotonated 1,1′-bis(*o*-carborane) anion [C_2_B_10_H_10_]_2_^2−^, which is the first example of a discrete molecular dicarbanion. These reactive carbanions are key intermediates in carbon vertex chemistry of carborane clusters.

## Abstract 2

Free three-dimensional carborane carbanions, which are inorganic siblings of deprotonated aryls with the “naked” anionic carbon atom are reported.

## Graphical abstract

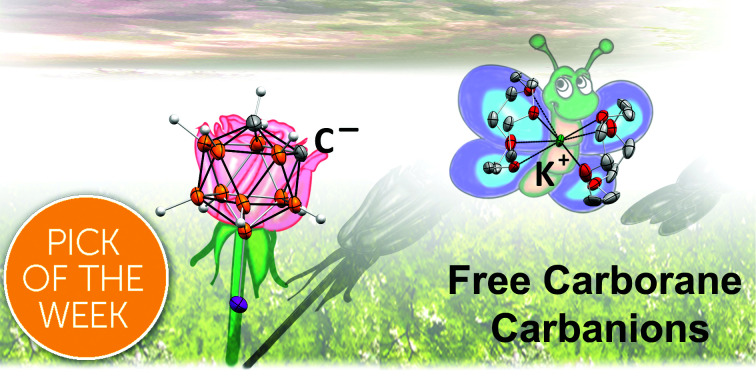

## Introduction

**Scheme 1:**
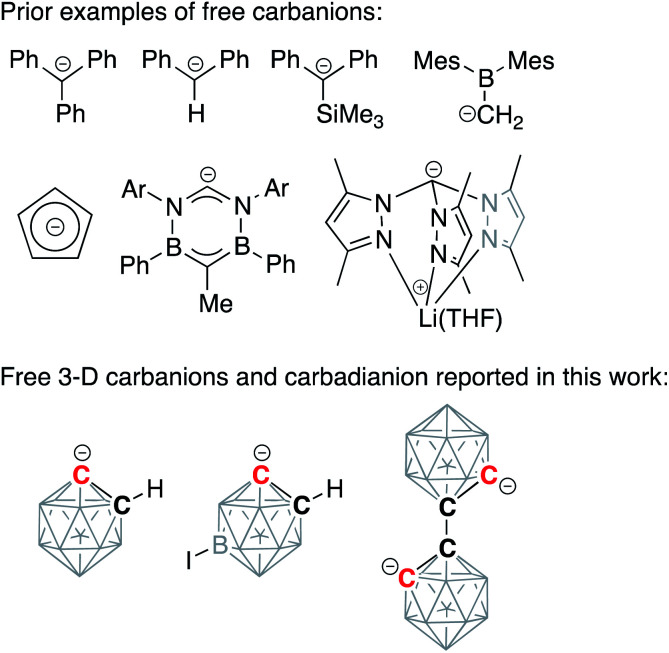
Examples of discrete carbanions isolated and structurally characterized in solid state from literature and in this work. Unlabeled cluster vertices represent BH units.

Alkali-metalated carbanions are reactive synthetic blocks, which exist as complex mixtures of aggregates and solvates.^1,2^ Organolithium reagents, which play an important role in organic synthesis, dominate the chemistry of these strong carbon-based nucleophiles, while sodium and potassium congeners remain relatively less studied.^3–6^ Group 1 organometallic compounds exhibit a variety of coordination modes, spanning from contact molecules with bridging non-classical covalent bonding to completely dissociated pairs of ions. These separated ion pairs, which display increased nucleophilicity due to an essentially uncompensated negative charge on the deprotonated carbon atom, have attracted significant attention from synthetic organometallic chemists. One successful strategy for the synthesis of carbanion-containing separated ion pairs, involving the use of crown ethers to capture metal cations, was pioneered by Power and co-workers for the synthesis of diarylmethyl and triarylmethyl anions.^7,8^ Subsequently, several free, discrete, “naked” carbanion centers have been isolated and structurally characterized, with the majority being based on sp^3^-hybridized carbon atoms and often being stabilized by the presence of silyl or aromatic substituents (Scheme 1).^9–14^ Importantly, separated ion pairs based on sp^2^-hybridized carbon atoms of aromatic arenes have not been yet isolated while the related free phenylide-like carbanions based on heterocyclic carbenes have been reported.^15^

In addition to organic reactions involving carbanions, some inorganic transformations rely upon negatively charged carbon nucleophiles. Icosahedral carboranes are chemically robust three-dimensional clusters built of carbon and boron atoms connected by delocalized intracluster bonds.^16^

The high degree of electron delocalization leads to the remarkable stability of carboranes and can be described as 3D aromaticity.^17–19^ The unusual steric and electronic properties of boron cluster derivatives have been utilized in coordination chemistry, catalysis, luminescent materials, metal–organic frameworks, polymers, medicine, and energy storage.^20–33^ The most commonly studied icosahedral anionic [CB_11_H_12_]^−^ and neutral C_2_B_10_H_12_ clusters contain relatively acidic C–H bonds (for example, p*K*_a_ of *ortho*-dicarbaborane is *ca.* 23).^16^ The major route for carbon vertex derivatization of these molecules relies on deprotonation of the carbon atom with strong bases (*e.g. n*-BuLi, i-PrMgCl, or KHMDS). Metalated carborane clusters synthesized in this manner are most of the time not isolated but utilized *in situ* in subsequent reactions with organic or inorganic electrophiles.^34–39^ Recently, Duttwyler and co-workers reported the synthesis and structural characterization of a lithiated anionic cluster Li[CB_11_H_11_]^−^ containing a lithium–carbon bond, which shed light on the structure of these nucleophilic intermediates and can be considered as a 3D analog of phenyllithium.^40^

In this work, we report the synthesis, structural, and spectroscopic characterization of deprotonated discrete carboranyl anions, including [C_2_B_10_H_11_]^−^, which, containing the “naked” carbanionic center, are the key intermediates in C-vertex functionalization of carboranes (Scheme 1, bottom). These reactive species have been considered elusive for a number of years, yet the careful utilization of crown ethers yielded stable dissociated salts of three-dimensional carbanions. The isolated discrete carboranyls can be considered as organomimetic inorganic three-dimensional analogs of the free phenyl anion.

## Results and discussion

**Scheme 2:**
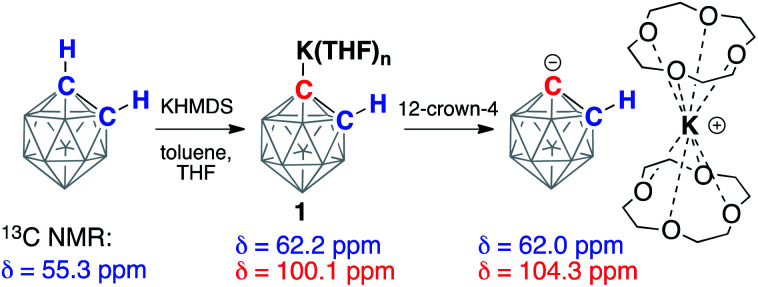
Deprotonation of *ortho*-C_2_B_10_H_12_ with potassium hexamethyldisilazide (KHMDS) and subsequent sequestration of the potassium cation by the crown ether. Chemical shifts from the signals in ^13^C NMR spectra from the carbon atoms of the parent and deprotonated clusters are listed. Unlabeled cluster vertices represent BH units.

*ortho*-Carborane (C_2_B_10_H_12_) contains two adjacent and weakly acidic C–H bonds (p*K*_a_*ca.* 23). The use of strong bases, such as alkyllithiums, has been reported to cause deprotonation of one or both carbon atoms of the carborane depending on the reaction stoichiometry.^34^ In our experiments, the addition of either one or two equivalents of potassium hexamethyldisilazide (KHMDS) to carborane in THF at room temperature resulted in the formation of the same single product according to ^13^C and ^11^B NMR spectroscopy. The ^13^C NMR spectrum of the product in THF exhibited signals from the carbon atoms of the cluster at 62.2 ppm corresponding to the protonated carbon atom and at 100.1 ppm corresponding to the metalated carbon atom. For comparison, lithiation of aryls leads to strong downfield shifts in ^13^C NMR spectra for metalated carbons.^41^ The signal from the remaining protonated carbon atom of the cluster in the ^13^C NMR spectrum correlates to the signal in the ^1^H NMR spectrum at 2.83 ppm. The ^11^B{^1^H} NMR spectrum of the product exhibited six signals, which is consistent with the *C*_s_ symmetry of the molecule. These observations suggest that the reaction of carborane and potassium hexamethyldisilazide leads to deprotonation of only one carbon atom and formation of KC_2_B_10_H_11_ (**1**) (Scheme 2).

Crown ethers have been demonstrated to assist in efficient ion-pair separation for a number of metal alkyls.^8^ The addition of two equivalents of 12-crown-4 to a solution of freshly prepared KC_2_B_10_H_11_ in THF led to a color change from pale-yellow to orange. The new band appeared at 442 nm in the absorption spectrum of **1** in THF upon the addition of 12-crown-4 (Fig. S-24 in ESI†). The ^13^C NMR spectrum of the reaction mixture at room temperature contained no signals attributable to the carborane carbon atoms, indicating the occurrence of an exchange process. Lowering the temperature of the sample to −15 °C led to the gradual appearance of two broadened signals at 62.0 ppm and 104.3 ppm (*cf.* 62.2 ppm and 100.1 ppm signals prior to the addition of the crown ether). The observed exchange process may occur due to coordination/dissociation of the potassium ion to the carborane, as well as proton exchange between deprotonated and protonated carbon atoms of the cluster when the potassium cation is complexed with crown ether. Notably, the proton exchange equilibrium between the singly deprotonated, doubly deprotonated, and fully protonated forms of *ortho*-carborane in ethereal solvents has been previously observed.^38^

**Fig. 1:**
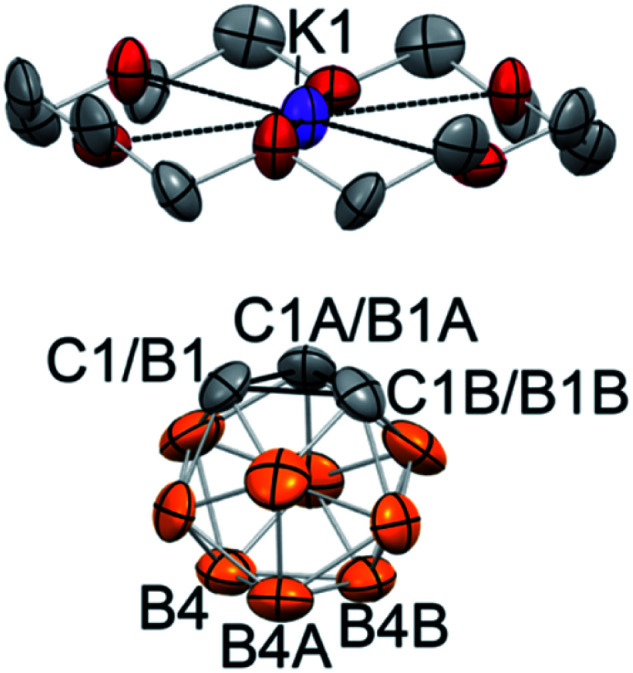
Displacement ellipsoid plot (50% probability) of [K(18-crown-6)][C_2_B_10_H_11_] (**2**). Hydrogen atoms have been omitted for clarity. Deprotonated carborane cluster is disordered by the crystal symmetry along its *C*_3_ axis. The closest potassium-cluster contact K1⋯C1/B1 is 3.586(1) Å.

The reaction between 18-crown-6, which exhibits a strong affinity to potassium cations, and KC_2_B_10_H_11_ with subsequent crystallization from the THF/toluene solvent mixture at −30 °C resulted in the formation of extremely air-sensitive orange single crystals. X-ray diffraction study revealed the structure of [K(18-crown-6)][C_2_B_10_H_11_] (**2**) (Fig. 1). Potassium cations are enclosed within the crown ether cavity by coordination to its six oxygen atoms. The deprotonated carborane anion and ligated potassium cations form alternating chains. In this rhombohedral crystal structure, the icosahedral carborane anion has crystallographic *C*_3v_ point symmetry and contains only four non-hydrogen cluster atoms in the asymmetric unit. Therefore, two carbon atoms and one boron atom of the cluster were disordered by the crystal symmetry among three positions. Nevertheless, the closest potassium-cluster contact was found to be 3.586(1) Å, which significantly exceeds the range for K–C bonds reported in the literature (2.75(3) to 3.247(3) Å).^42–44^ The determination of this structure along with ^13^C NMR spectroscopy data led us to believe that the complete ion separation is possible for the metalated carborane cluster, and that the discrete deprotonated carborane anion can be isolated and properly characterized if its symmetry were lowered to prevent crystallographic disorder. Thus, we utilized 9-iodo-*ortho*-carborane that contains one iodine atom attached to a boron atom of the cluster on the side that is opposite to carbon atoms.^45^

The reaction of 9-iodo-*ortho*-carborane and KHMDS in toluene, analogously to the unsubstituted *ortho*-carborane, resulted in the formation of monometalated species. Introduction of one iodine substituent lowers the symmetry of the cluster and renders two carbon atoms inequivalent, thus we observed the signals from two isomers of monodeprotonated clusters in *ca.* 2 : 1 ratio in NMR spectra. The ^13^C NMR spectrum of K[C_2_B_10_H_10_I] in THF contained two sets of pairs of signals from the carbon atoms of the cage: one, corresponding to the protonated *C*(H), at 58.4/61.4 ppm (major/minor isomer), and another, corresponding to the deprotonated *C*(K), at 100.8/86.6 ppm (major/minor isomer). The assignment of signals from metalated *C*(K) and protonated *C*(H) carbon atoms was corroborated by the DEPT-135 spectrum. Note that the inequivalent protonated carbon atoms of the parent 9-iodo-*ortho*-carborane exhibit signals at 50.2 ppm and 54.6 ppm in the ^13^C NMR spectrum.

**Fig. 2:**
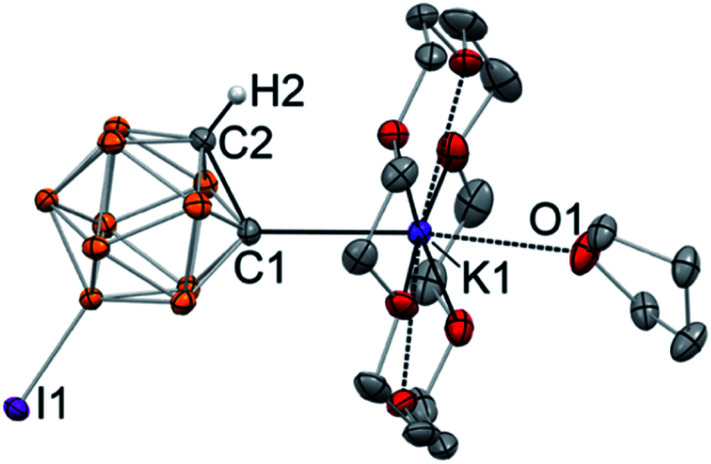
Displacement ellipsoid plot (50% probability) of [K(18-crown-6)(THF)][C_2_B_10_H_10_I] (**3**). Hydrogen atoms except H2 have been omitted for clarity. Selected bond distances (Å): K1–C1 = 2.978(1), C1–C2 = 1.682(1).

Addition of 18-crown-6 to the solution of K[C_2_B_10_H_10_I] in THF and subsequent crystallization from the THF/toluene mixture at −30 °C resulted in the formation of single crystals. The structure of the metalated 9-I-*ortho*-carborane was elucidated with the use of single crystal X-ray crystallography (Fig. 2). The crystal structure revealed that, as expected, 9-I-*ortho*-carborane is metalated with one potassium atom with the carbon–metal bond distance of 2.978(1) Å, which is on the longer end of the range of known C–K bond distances. The potassium atom is also bound to the crown ether and the THF ligand. The intracluster C1–C2 bond length in the monoanion is 1.682(1) Å, which is slightly longer than that in the protonated C_2_B_10_H_11_I (1.636(8) Å).^46^ The remaining protonated carbon atom of the cluster is located in the *para*-position opposite to the iodinated boron atom B9.

In order to enforce the complete ion separation, we tested the hypothesis that the coordination of two smaller crown ether ligands will preclude potassium from additional bonding to anionic carbon atoms of carborane. The addition of two equivalents of 12-crown-4 to a solution of K[C_2_B_10_H_10_I] in THF at room temperature led to an immediate color change from yellow to intense orange. The ^13^C NMR spectrum of the reaction mixture at −15 °C contained a pair of signals corresponding to protonated carbon atoms at 58.6/61.4 ppm (major/minor isomer) and another pair corresponding to deprotonated carbon atoms at 105.4/90.7 ppm (major/minor isomer). Notably, the signals from the anionic carbon atom shifted further downfield upon the addition of crown ether.

**Fig. 3:**
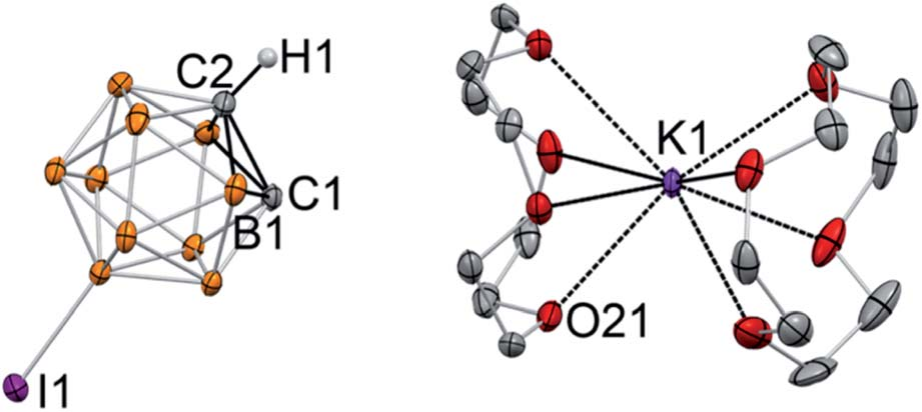
Displacement ellipsoid plot (50% probability) of [K(12-crown-4)_2_][C_2_B_10_H_10_I] (**4**). Note the lack of C⋯K contact and the complete ion separation. Only a half of the asymmetric unit is shown. Hydrogen atoms on the boron atoms of the cluster and carbon atoms of the crown ether ligand have been omitted for clarity. Selected interatomic distances (Å): K1⋯C1 = 6.019(3), C1–C2 = 1.677(6), C1–B1 = 1.717(7), C2–B1 = 1.671(8). The analogous distances for another ion pair in the asymmetric unit are K2⋯C11 = 5.962(5), C11–C12 = 1.674(6), C11–B11 = 1.709(7), C12–B11 = 1.678(7).

Single crystals of the product were grown at −30 °C from a THF/toluene solvent mixture. X-ray diffraction study revealed the complete separation of the cation and anion in [K(12-crown-4)_2_][C_2_B_10_H_10_I] (**4**) (Fig. 3). The closest K⋯C contacts in the structure are 5.962(5) Å and 6.019(3) Å, which are out of range for any significant bonding. Gratifyingly, the potassium cation is coordinated to eight oxygen atoms of two crown ether ligands and does not have any additional interatomic interactions. No disorder was observed in this structure due to the lower symmetry of the iodinated carborane cluster. The high quality of the diffraction data along with the presence of the iodine substituent opposite to one of the carbon atoms of the cage allowed us to unambiguously assign carbon and boron atoms of the free cluster anion. Furthermore, the hydrogen atom of the remaining C–H bond was clearly located in difference Fourier maps, while there was no electron density observed in the vicinity of the deprotonated carbon atom. The intracluster C1–C2 bond length in the free discrete [C_2_B_10_H_10_I]^−^ anion is 1.677(6) Å, which is similar to that for its metalated form K[C_2_B_10_H_10_I] (Fig. 2). Notably, the presence of the carbanion alters interatomic distances within the cluster, with the carbon–boron bonds for the deprotonated carbon atom being significantly longer than those for the protonated one. For example, boron atom B1 which is connected to both C1 and C2(H1) exhibits a longer bond length (B1–C1 = 1.717(7) Å) than that for the bond to the protonated C2 atom (B1–C2 = 1.671(8) Å).

The structure of [K(12-crown-4)_2_][C_2_B_10_H_10_I] is the first example of an unambiguously spectroscopically and structurally characterized free, discrete deprotonated carborane anion with the “naked” carbanion center. Prior to this work, the free carboranyl anion [C_2_B_10_H_11_]^−^ was regarded as elusive and “unlikely to be isolated”.^47^ Deprotonated carboranyls have been inferred to exist when paired with organic cations, however, in some cases these compounds were found to instead contain [C_4_B_20_H_23_]^−^, which is the product of the nucleophilic attack of the mono deprotonated carborane cluster on its neutral parent cage.^47,48^ The boron-perhalogenated H_2_C_2_B_10_I_10_, which possesses significantly acidic C–H bonds (reported p*K*_a_ is between 2.9 and 4.7), was deprotonated and paired with the [N(PPh_3_)_2_]^+^ cation; however, the putative PPN salt has been characterized only by mass-spectrometry.^49^ In this work, we demonstrated that “naked” deprotonated carboranyl anions can be isolated with the use of crown ethers. Given the high degree of electron delocalization in carboranes, the discrete organomimetic anions reported herein are also related to the still elusive aromatic free carbanions. Attempts of their synthesis and isolation have been reported, including unsuccessful utilization of crown ethers with lithiated aryls.^41^

**Fig. 4:**
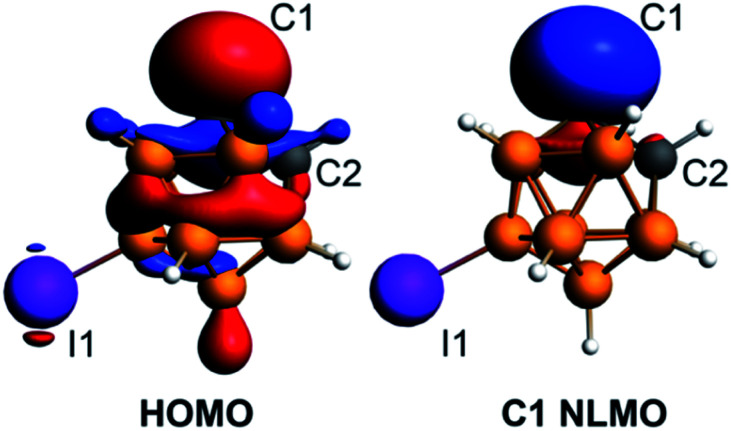
Plots of the HOMO and the C1 carbon atom lone pair NLMO for the [C_2_B_10_H_10_I]^−^ anion at the isosurface value of 0.035.

The density functional theory calculations of the electronic structure of **4** utilizing ADF^50^ with the hybrid PBE0 functional and the ATZP basis set^51^ confirmed the strongly nucleophilic character of the isolated discrete carbanion. The HOMO of the [C_2_B_10_H_10_I]^−^ anion is largely located at the deprotonated carbon atom with some delocalization throughout the cluster (Fig. 4). The next lower energy occupied orbital HOMO−1 is localized at the iodine substituent. The Natural Bonding Orbital (NBO) analysis^52^ of the localized lone pair orbital on C1 shows that is composed from s (33%) and p (67%) atomic orbitals (the composition of this carbon-based hybrid orbital is sp^2^). The Natural Localized Molecular Orbital (NLMO) analysis demonstrated that the lone pair orbital on the deprotonated carbon atom is by 92% composed of the corresponding parent lone pair NBO on C1 (Fig. 4).

**Scheme 3:**
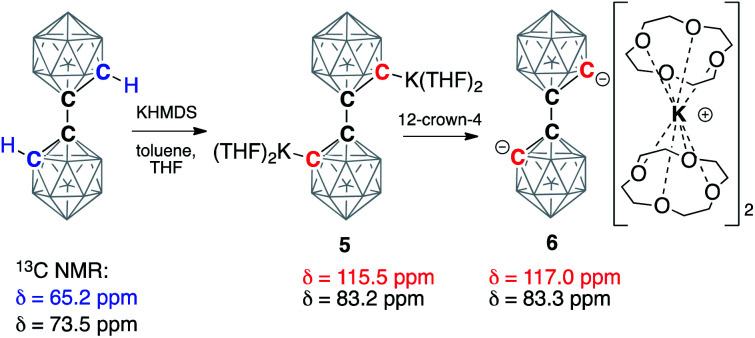
Double deprotonation of 1,1′-bis(*o*-carborane) (C_2_B_10_H_11_)_2_ with potassium hexamethyldisilazide (KHMDS) and subsequent sequestration of potassium cations by the crown ether. Chemical shifts of the signals in the ^13^C NMR spectra from carbon atoms of the parent and deprotonated clusters are listed. Unlabeled cluster vertices represent BH units.

Next, we moved our attention to the study of 1,1′-bis(*o*-carborane) (C_2_B_10_H_11_)_2_, which is an analog of biphenyl, being composed of two carborane clusters bound together through a single C–C bond, thus leaving two C–H bonds amenable to deprotonation.^53,54^ Analogously to carboranes, metalation of biscarboranes is the main route for their carbon vertex functionalization.^55–57^ We found that the addition of two equivalents of KHMDS to the solution of biscarborane leads to its complete deprotonation as evidenced by the appearance of two new signals in the ^13^C NMR spectrum in THF at 83.2 ppm, corresponding to the intercluster C–C bond, and 115.5 ppm, corresponding to the metalated carbon atoms (*cf.* 65.2 ppm for *C*(H) and 73.5 ppm for *C*(C) carbon atoms for the parent neutral biscarborane) (Scheme 3). The addition of four equivalents of 12-crown-4 to this solution led to a slight shift of the signal from the *C*(C) carbon atom to 83.3 ppm and a larger shift for the signal from the deprotonated anionic carbon to 117.0 ppm. Notably, both resonances were sharp at room temperature, indicating the absence of proton exchange equilibria in this system as the doubly deprotonated form lacks reasonably acidic hydrogen atoms.

Crystallization from a THF/toluene solvent mixture of the deprotonated biscarborane with and without added crown ether resulted in the formation of single crystals suitable for X-ray diffraction study. With no crown ether present, the structure of [K(THF)_2_]_2_(C_2_B_10_H_10_)_2_ (**5**) was determined (see ESI† page S-29 for the figure and details). Potassium cations in **5** are coordinated to the carbon atoms of biscarborane. One of the cations is strongly bound to both deprotonated carbons of two cages with the bond distances of K2–C1 2.997(1) Å and K2–C4 2.843(1) Å. Another potassium cation is only weakly bound to one of the cluster carbons with the K1–C1 bond length of 3.175(1) Å. The metalated carbon atoms of the two clusters are in a *cis*-orientation relative to each other.

**Fig. 5:**
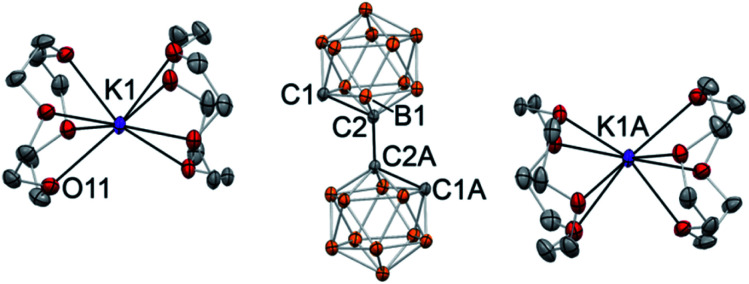
Displacement ellipsoid plot (50% probability) of [K(12-crown-4)_2_]_2_(C_2_B_10_H_10_)_2_·4(THF) (**6**). Note the lack of C⋯K contact and the complete ion separation. Only a half of the asymmetric unit is shown. Hydrogen atoms on boron atoms of the cluster and carbon atoms of the crown ether ligand have been omitted for clarity. Crystallization solvent molecules (THF) are not shown. Selected interatomic distances (Å): K1⋯C1 = 6.283(1), C1–C2 = 1.712(1), C1–B1 = 1.724(2), C2–B1 = 1.698(2), C2–C2A = 1.532(2) Å.

In the case of crown ether addition, orange single crystals of [K(12-crown-4)_2_]_2_(C_2_B_10_H_10_)_2_·4(THF) (**6**) were obtained (Fig. 5). As we envisaged, potassium ions are entirely enclosed by pairs of crown ether ligands with no metal–carbon bonds (the shortest C1⋯K1 distance is 6.283(1) Å; the shortest distance between the cation and the cluster is at 4.664(2) Å between the potassium and one of the boron atoms). The 1,1′-bis(*o*-carboranyl) dianion, featuring two “naked” carbanionic centers in a *trans*-orientation relative to each other, exhibits the elongated intracluster C1–C2 distance of 1.712(1) Å and the intercluster C2–C2A bond length of 1.532(2) Å. The distance between boron atom B1 of the cluster and the deprotonated anionic carbon atom B1–C1 is 1.724(2) Å, while its bond length to the carbon atom connecting to the second cage B1–C2 is significantly shorter at 1.698(2) Å. The completely dissociated ion pair [K(12-crown-4)_2_]_2_(C_2_B_10_H_10_)_2_ is the first example of a molecular doubly deprotonated free carbanion with a dinegative charge. DFT calculations indicate that HOMO and HOMO−1 of the free biscarboranyl dianion are localized on the deprotonated carbon atoms (see Fig. S-23 in ESI† for details).

**Scheme 4:**
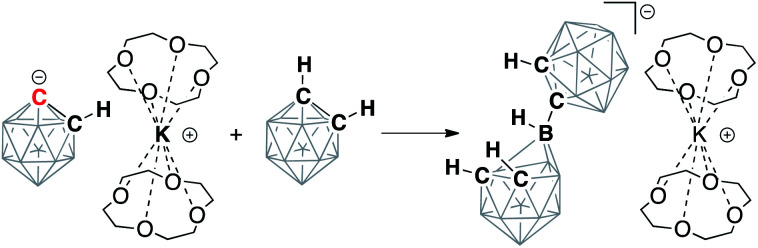
Reaction of the strongly nucleophilic deprotonated free carboranyl anion with the parent protonated carborane cluster and the formation of the two-cage anion [C_4_B_20_H_23_]^−^. Unlabeled cluster vertices represent BH units.

The formation of a “naked” carbanion within the completely separated ion pair in the presence of crown ether ligands is expected to lead to an increase in its nucleophilic character. Addition of *ortho*-carborane to the solution of [K(12-crown-4)_2_][C_2_B_10_H_11_] led to the immediate formation of a single product, the previously characterized [C_4_B_20_H_23_]^−^ anion, at room temperature according to ^11^B and ^13^C NMR spectroscopy (Scheme 4). This product is formed upon nucleophilic attack of the discrete [C_2_B_10_H_11_]^−^ anion on neutral C_2_B_10_H_12_. Its formation has also been previously reported in the reactions of *ortho*-carborane with sterically hindered N-heterocyclic carbenes.^47^ The gradual formation of [C_4_B_20_H_23_]^−^ has also been observed in solutions of the lithiated carborane Li[C_2_B_10_H_11_] in THF upon prolonged standing (days) at room temperature or at reflux overnight in the presence of alkali metal halides.^38^ The facile formation of this product from the reaction of [C_2_B_10_H_11_]^−^ and C_2_B_10_H_12_ at room temperature demonstrates the dramatic enhancement of nucleophilicity of the carboranyl anion upon complete separation of the potassium cation from the anionic carbon. Notably, this rapid formation of the dimeric [C_4_B_20_H_23_]^−^ cluster was not observed in the THF solution of [K(12-crown-4)_2_][C_2_B_10_H_11_] upon standing, as it apparently requires the presence of the neutral C_2_B_10_H_12_ cluster, or, in other words, nucleophilic bimolecular decomposition of discrete [C_2_B_10_H_11_]^−^ anions is presumably precluded by their negative charges.

## Conclusions

The utilization of crown ether ligands facilitated the isolation and structural characterization of “naked” carborane cluster carbanions. This strategy also resulted in the synthesis of the first dianionic deprotonated biscarborane cluster, containing two carbanionic centers. The obtained completely separated ion pairs feature non-planar carbanions stabilized by the three-dimensional boron cluster framework. These electron-rich inorganic nucleophiles have been regarded as elusive species prior to this work, and they are related to yet unknown discrete aromatic phenyl anions. The synthesis of reactive free anionic carboranyl fragments provides an insight into the structure of intermediates in carbon vertex derivatization and opens a way for broadening the scope of functionalized molecular boron clusters.

## Data availability

CCDC 2078641–2078646 contain the supplementary crystallographic data for this paper. These data can be obtained free of charge *via*www.ccdc.cam.ac.uk/data_request/cif, or by emailing data_request@ccdc.cam.ac.uk, or by contacting The Cambridge Crystallographic Data Centre, 12 Union Road, Cambridge CB2 1EZ, U.K.; fax: +44 1223 336033.

## Author contributions

H. D. A. C. J. and M. M. R. designed experiments and carried out spectroscopic and synthetic work; P. J. P. and H. D. A. C. J. designed and carried out NMR spectroscopy experiments; M. D. S. carried out crystal structure determination and analysis; D. V. P. conceived the project, provided guidance, and carried out theoretical calculations. All authors contributed to the writing and editing of the manuscript.

## Conflicts of interest

There are no conflicts to declare.

## Supplementary Material

SC-012-D1SC02252K-s001

SC-012-D1SC02252K-s002
