# Supplementary material for: Free three-dimensional carborane carbanions
Source: Chem Sci. 2021 Jun 30;12(31):10441–7. doi: 10.1039/d1sc02252k (PMC8356735; doi:10.1039/d1sc02252k)
Supplement: SC-012-D1SC02252K-s001 [file SC-012-D1SC02252K-s001.pdf]

Electronic Supplementary Material (ESI) for Chemical Science.
This journal is © The Royal Society of Chemistry 2021

Electronic Supplementary Information (ESI)

Free Three-Dimensional Carborane Carbanions

H. D. A. Chathumal Jayaweera,[a] Md. Mamdudur Rahman,[a] Perry J. Pellechia,[a] Mark D. Smith,[a] and Dmitry V. Peryshkov*[a]

Department of Chemistry and Biochemistry, University of South Carolina, 631 Sumter St., Columbia, South Carolina 29208, United States

Table of Contents

Experimental details	S-2
NMR spectra of K[$C_2B_{10}H_{11}$] (**1**) in THF	S-4
NMR spectra of [K(12-crown-4)$_2$][$C_2B_{10}H_{11}$] in THF	S-7
NMR spectra of K[$C_2B_{10}H_{10}I$] and [K(12-crown-4)$_2$][$C_2B_{10}H_{10}I$] (**4**) in THF	S-10
NMR spectra of $K_2(C_2B_{10}H_{10})_2$ and [K(12-crown-4)$_2$]$_2$($C_2B_{10}H_{10}$)$_2$ (**6**) in THF	S-15
X-ray structure determination of [K(18-crown-6)][$C_2B_{10}H_{11}$] (**2**)	S-21
X-ray structure determination of [K(18-crown-6)(THF)][$C_2B_{10}H_{10}I$] (**3**)	S-24
X-ray structure determination of [K(12-crown-4)$_2$][$C_2B_{10}H_{10}I$] (**4**)	S-26
X-ray structure determination of [K(THF)$_2$]$_2$($C_2B_{10}H_{10}$)$_2$ (**5**)	S-29
X-ray structure determination of [K(12-crown-4)$_2$]$_2$($C_2B_{10}H_{10}$)$_2$·4(THF) (**6**)	S-31
X-ray structure determination of [K_2(18-crown-6)$_2$($C_4B_{20}H_{20}$)]·(C_7H_8)$_3$ (**7**)	S-33
Computational details	S-36
Absorption spectra of $KC_2B_{10}H_{11}$ and [K(12-crown-4)]$_2$[$C_2B_{10}H_{11}$] in THF	S-41

Experimental details.

All synthetic manipulations were carried out either in a nitrogen-filled VAC drybox or on a dual manifold Schlenk-style vacuum line. The solvents were sparged with nitrogen, passed through activated alumina, and stored over activated 4 Å Linde-type molecular sieves. Benzene-d^6 was degassed and stored over activated 4 Å Linde-type molecular sieves. NMR spectra were recorded in THF containing traces of C_6D_6 using Varian spectrometers at 400 (^{1}H), 100 (^{13}C), 128 (^{11}B) MHz, reported in δ (parts per million) and referenced to the residual ^{1}H/^{13}C signals of the deuterated solvent or an external $BF_3(Et_2O)$ (^{11}B(δ): 0.0 ppm) standard. *J* values are given in Hz.

1,1′-bis(*o*-carborane)[1], and 9-I-*ortho*-carborane[2] were prepared according to the previously reported literature procedures. *Ortho*-carborane $C_2B_{10}H_{12}$ (Boron Specialties), 18-crown-6 ether, 12-crown-4 ether, and potassium hexamethyldisilazide (KHMDS, 0.7 M solution in toluene) were used as received.

Synthesis of [K(12-crown-4)$_2$][$C_2B_{10}H_{10}I$] (4)

9-I-*ortho*-carborane (60 mg; 0.222 mmol) was dissolved in toluene (4 mL) in a 20 mL vial. Potassium hexamethyldisilazide (KHMDS) (0.7 M in toluene, 0.66 mL; 0.466 mmol, 2.1 equiv) was added to the 9-I-*ortho*-carborane solution dropwise and stirred overnight at room temperature. Hexane was added to the resulting pale-yellow color solution and the volatiles were removed under vacuum to yield a pale-yellow solid. The solid was washed with toluene (1 mL × 2) to remove any impurities and dried thoroughly. The solid was re-dissolved in THF (2 mL) and 12-crown-4 ether (82 mg; 0.466 mmol, 2.1 equiv) was added dropwise with the color change to intense yellow/orange. Toluene (1 mL) was added to the solution. Air-sensitive orange crystals formed upon standing in the freezer at −30 ºC. Yield: 105 mg, 72%

^{13}C (THF, −15 ºC) major isomer: δ 105.4 (carborane $C^{\ominus}$), 58.6 (carborane *C*-H); minor isomer: 90.7 (carborane $C^{\ominus}$), 61.4 (carborane *C*-H); 68.0 (12-crown-4).

Synthesis of [K(12-crown-4)$_2$]$_2$($C_2B_{10}H_{10}$)$_2$ (6)

1,1′-bis(*o*-carborane) (30 mg; 0.105 mmol) was dissolved in toluene (4 mL) in a 20 mL vial. Potassium hexamethyldisilazide (KHMDS) (0.7M in toluene, 0.32 mL; 0.220 mmol, 2.1 equiv) was added to the solution dropwise and stirred overnight at room temperature. A pale-yellow color turbid solution was observed right after the addition of the base. The resulting solution was dried under vacuum to remove the volatiles to obtain a pale-yellow solid. The solid was washed with toluene (1mL × 2) and dried. The solid was re-dissolved in THF (2 mL) producing an intense yellow solution. 12-crown-4 ether (76 mg; 0.430 mmol, 4.1 equiv) was added dropwise, resulting in the color change to orange. Toluene (1 mL) was added

to the solution. Air-sensitive dark orange microcrystals formed upon standing in the freezer at −30 ºC. Yield: 59 mg, 63%.

^{13}C (THF): δ 117.0 (biscarborane $C^{\ominus}$), 83.3 (biscarborane *C*-*C*), 68.4 (12-crown-4)

Synthesis of [K(12-crown-4)$_2$][$C_2B_{10}H_{11}$]

Ortho-carborane (50 mg; 0.347 mmol) was dissolved in toluene (4 mL) in a 20 mL vial. Potassium hexamethyldisilazide (KHMDS) (0.7 M in toluene, 1.04 mL; 0.729 mmol, 2.1 equiv) was added to the *ortho*-carborane solution dropwise and stirred overnight at room temperature. The volatiles were removed under vacuum from the resulting turbid pale-yellow color solution to yield a pale-yellow solid. The solid was washed with toluene (1mL × 2) to remove any impurities and dried thoroughly. The solid was re-dissolved in THF (2 mL) and 12-crown-4 ether (128 mg; 0.729 mmol, 2.1 equiv) was added dropwise with color change from yellow color to orangish-yellow. Air-sensitive orange single crystals formed upon standing in the freezer at −30 ºC.

^{13}C (THF, −15 ºC): δ 104.3 (carborane $C^{\ominus}$), 62.0 (carborane *C*-H), 66.6 (12-crown-4).

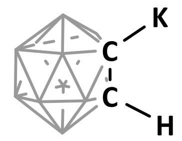

1

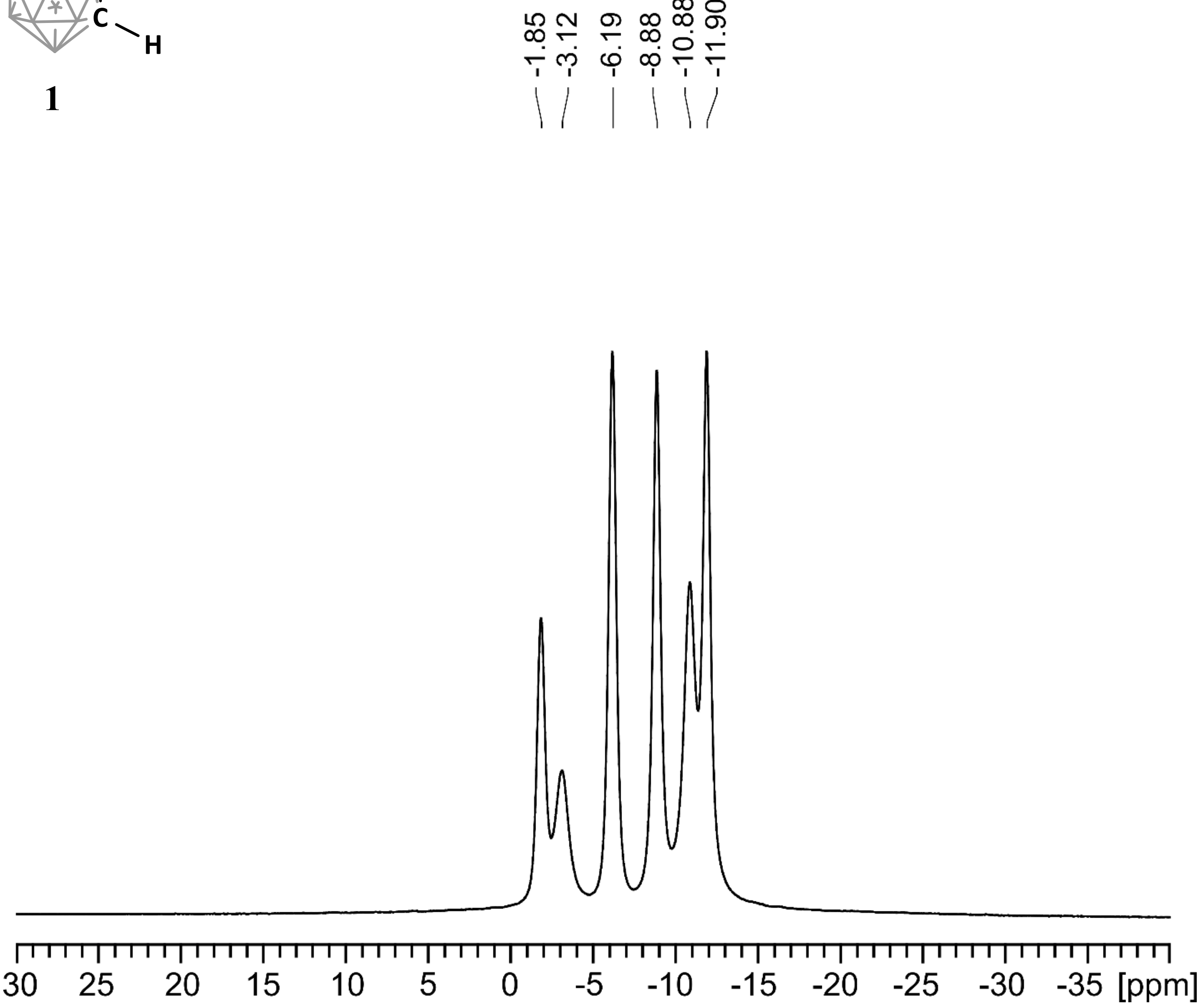

Figure S-1. The $^{11}B\{^{1}H\}$ NMR spectrum of $C_2B_{10}H_{11}K$ in THF at room temperature.

$^{11}B\{^{1}H\}$ (THF): δ –1.8, –3.1, –6.2, –8.9, –10.9, –11.9.

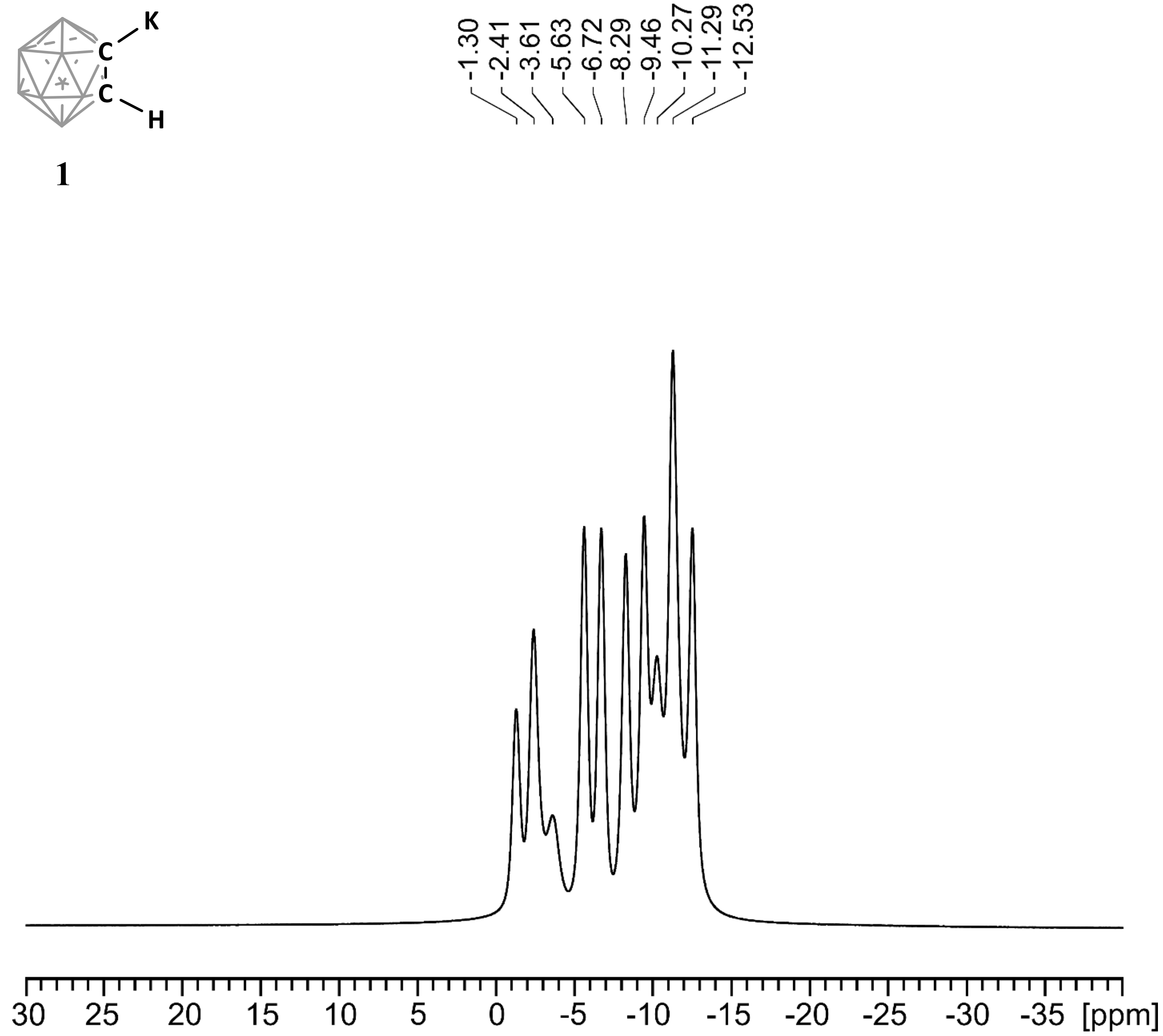

Figure S-2. The ^{11}B NMR spectrum of $C_2B_{10}H_{11}K$ in THF at room temperature.

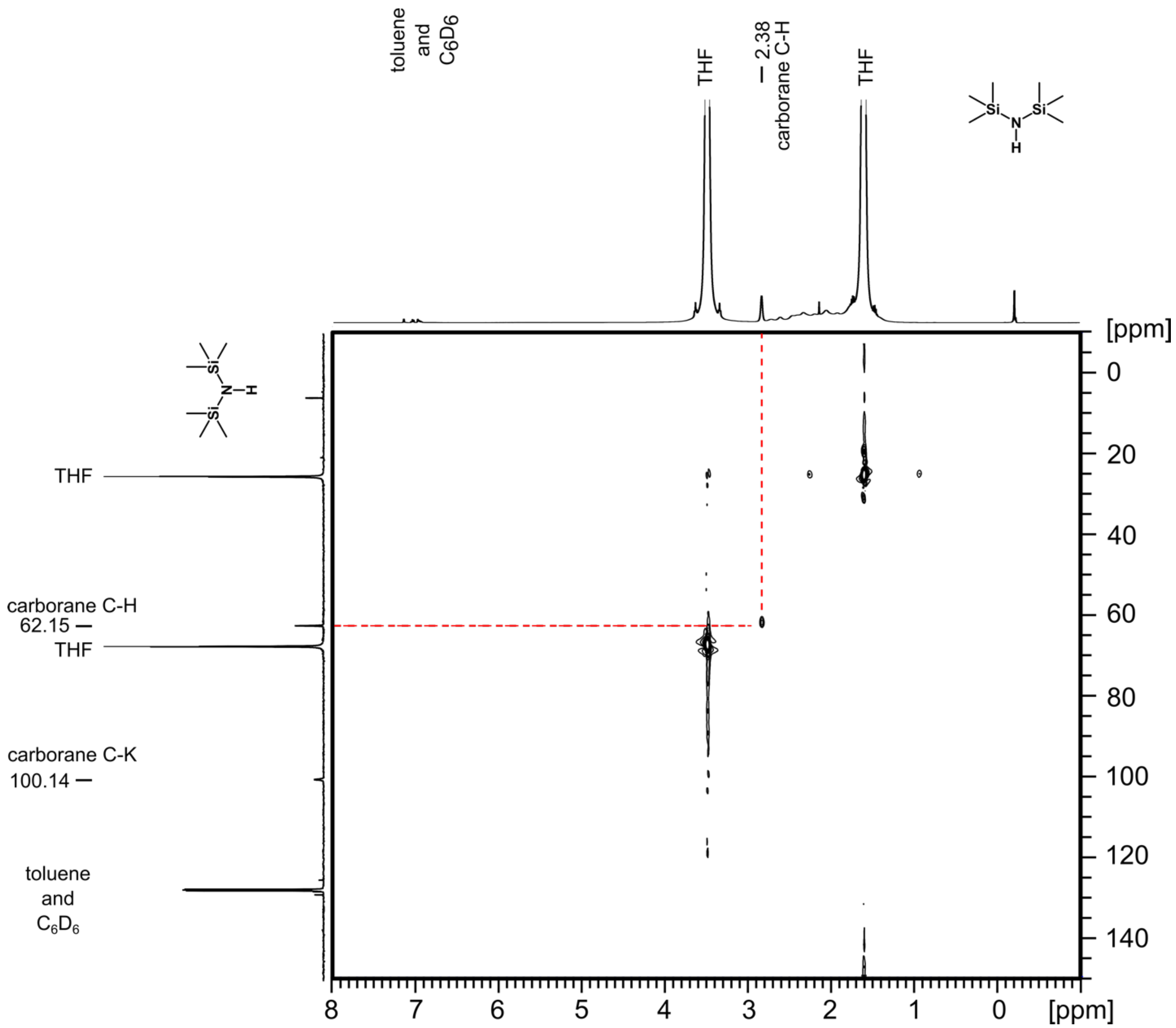

Figure S-3. The ^{13}C-^{1}H COSY NMR spectrum of $C_2B_{10}H_{11}K$ in THF at room temperature.

^{13}C (THF): δ 100.1 (*C*-K), 62.2 (*C*-H). ^{1}H (THF): δ 2.38 (C-*H*)

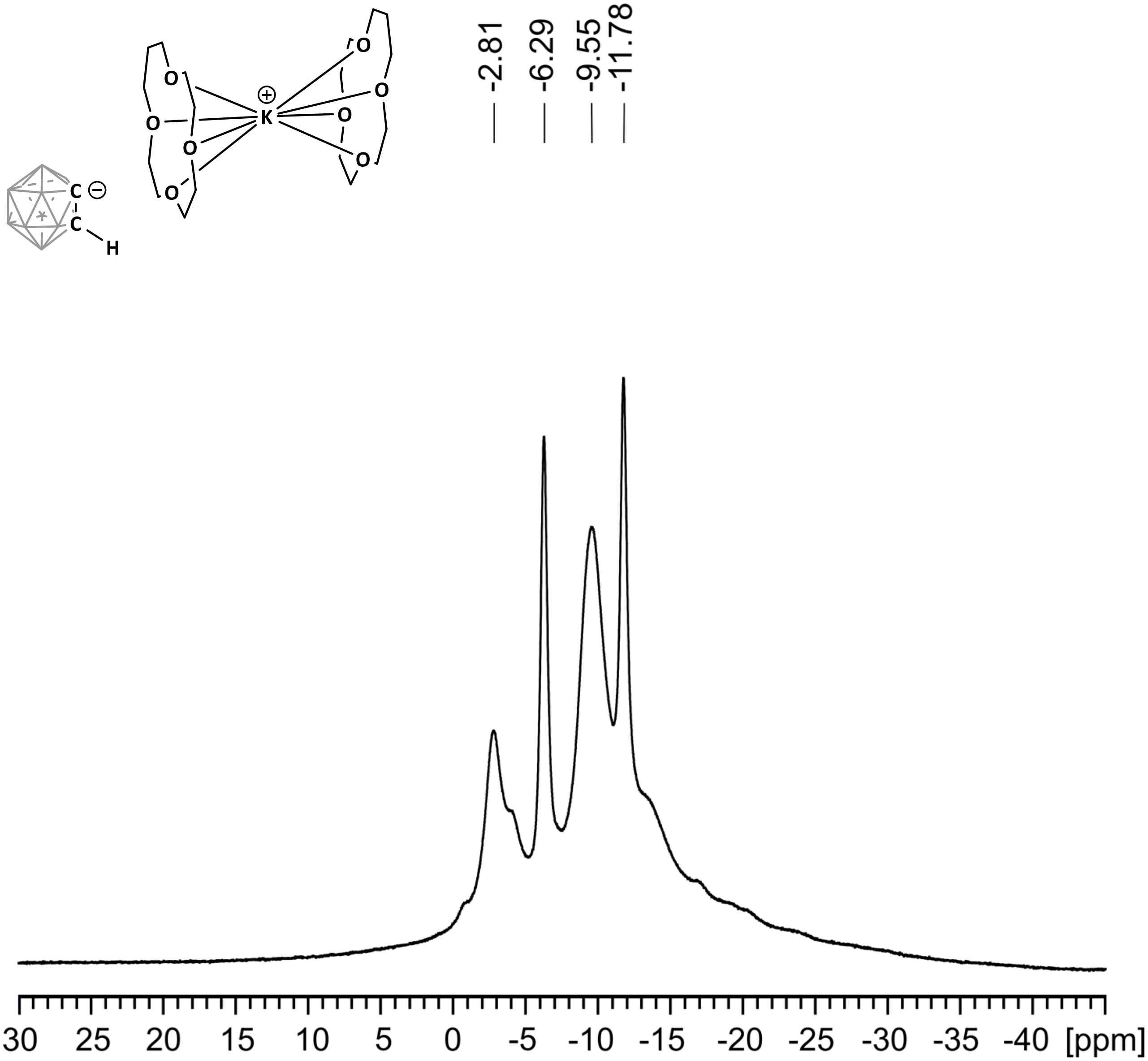

Figure S-4. The $^{11}B\{^{1}H\}$ NMR spectrum of $[K(12\text{-crown-}4)_2][C_2B_{10}H_{11}]$ in THF at room temperature.

$^{11}B\{^{1}H\}$ (THF): δ –2.8, –6.3, –9.6, –11.8

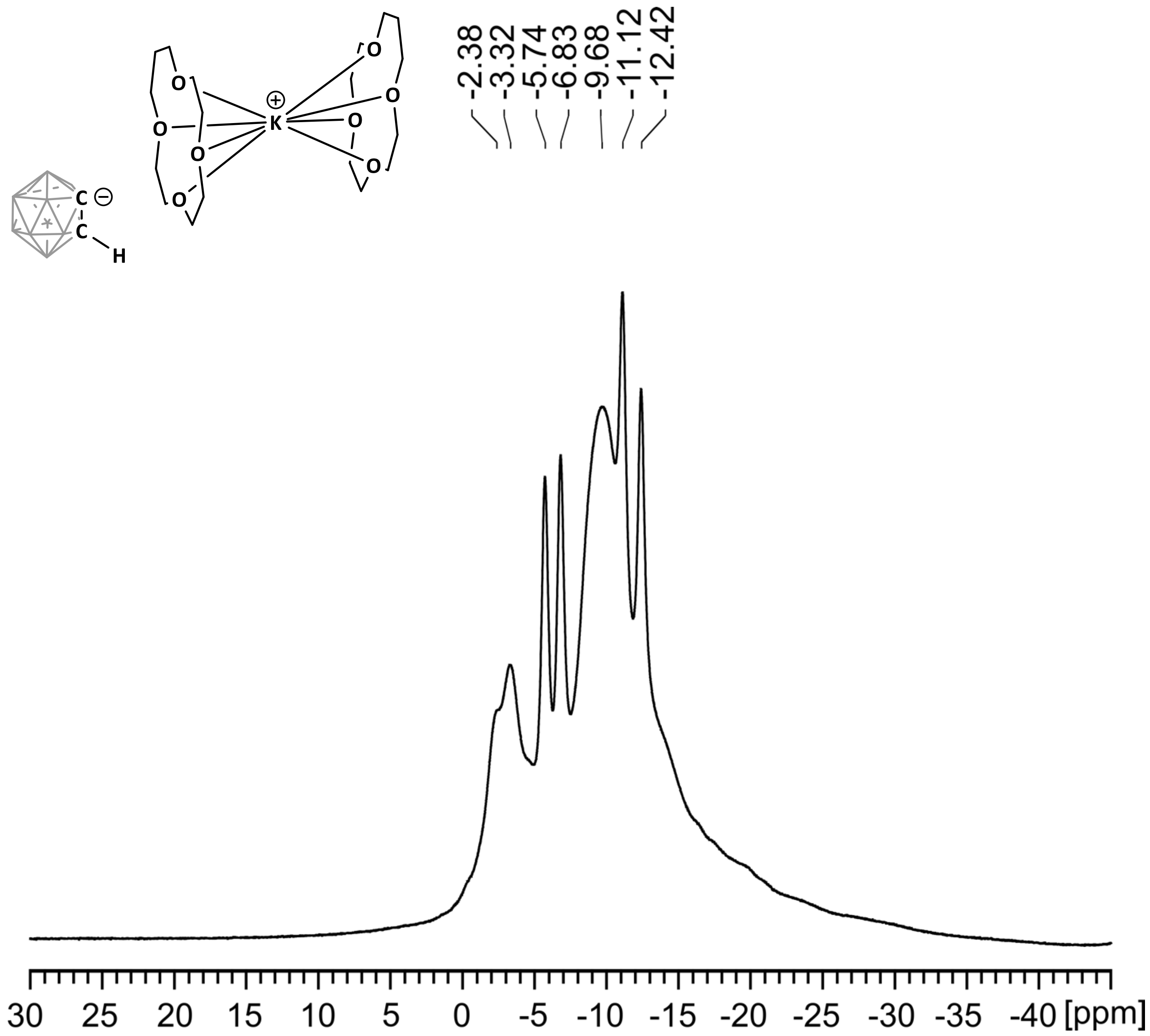

Figure S-5. The ^{11}B NMR spectrum of $[K(12\text{-crown-}4)_2][C_2B_{10}H_{11}]$ in THF at room temperature.

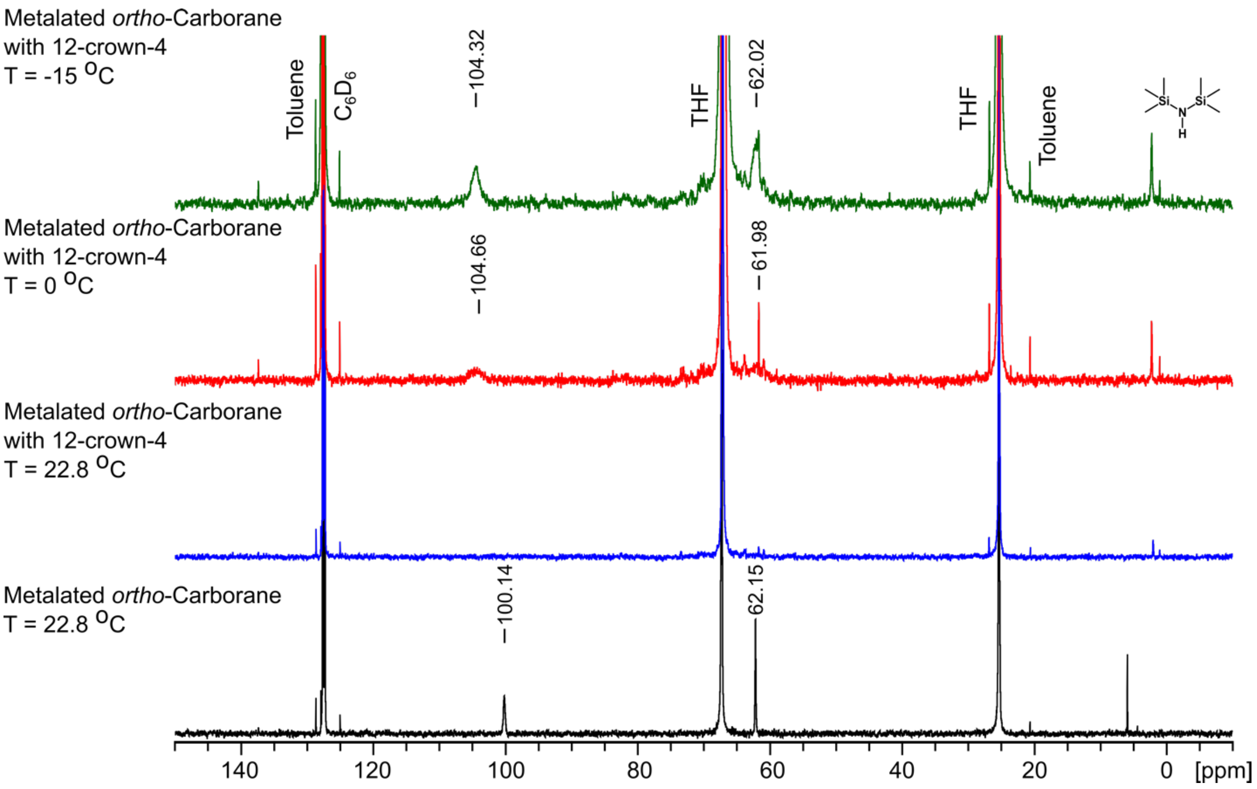

Figure S-6. Variable temperature ^{13}C NMR spectra of $[K(12\text{-crown-}4)_2][C_2B_{10}H_{11}]$ in THF. Note the gradual appearance of the signals from the protonated and deprotonated carbon atom of the cluster upon lowering temperature of the sample.

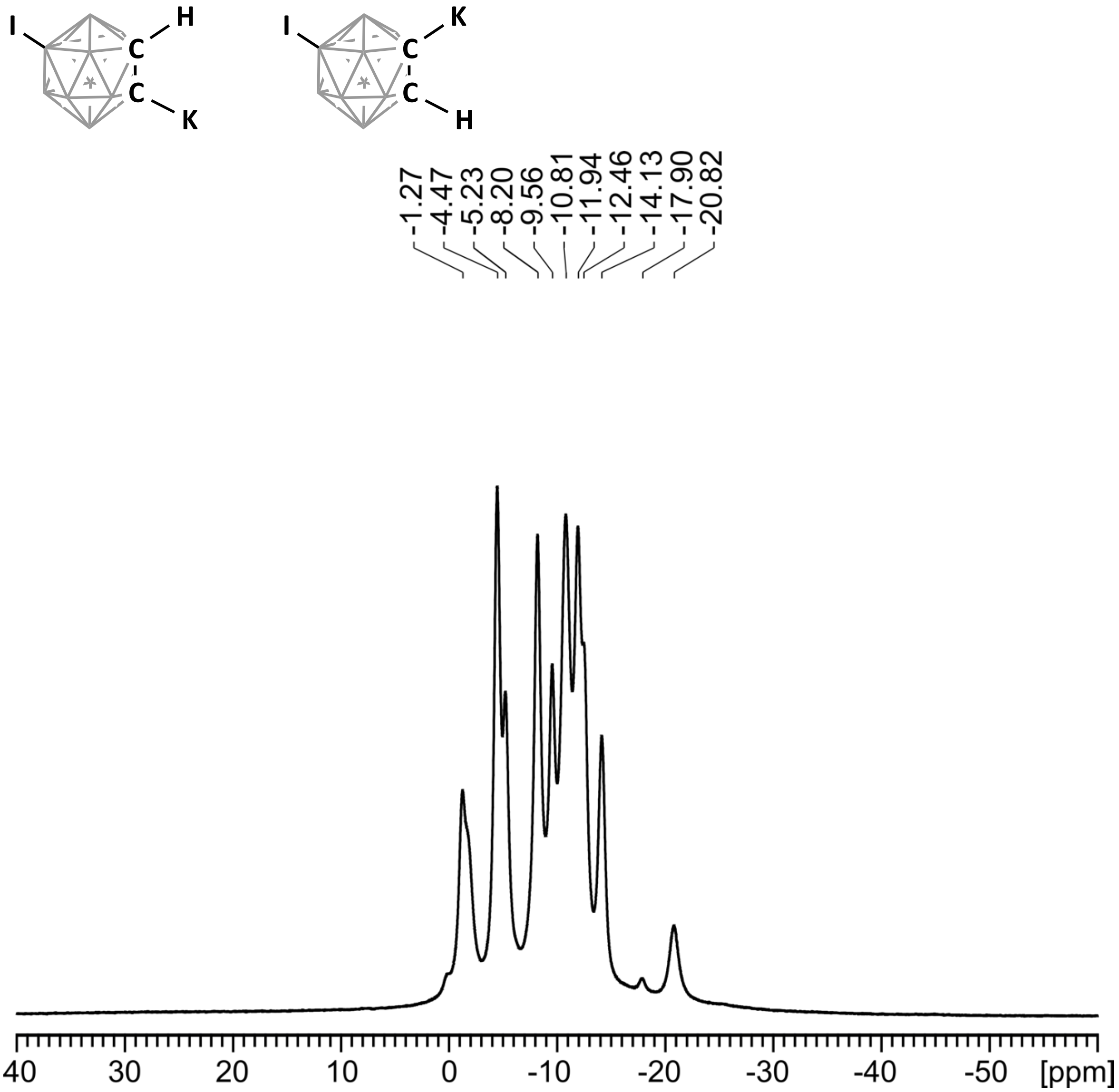

Figure S-7. The $^{11}B\{^{1}H\}$ NMR spectrum of $K[C_2B_{10}H_{10}I]$ in THF at room temperature. Note that two isomers of the monodeprotonated cluster were observed.

$^{11}B\{^{1}H\}$ (THF): δ -1.27, -4.47, -5.23, -8.20, -9.56, -10.81, -11.94, -12.46, -14.13, -17.90, -20.82

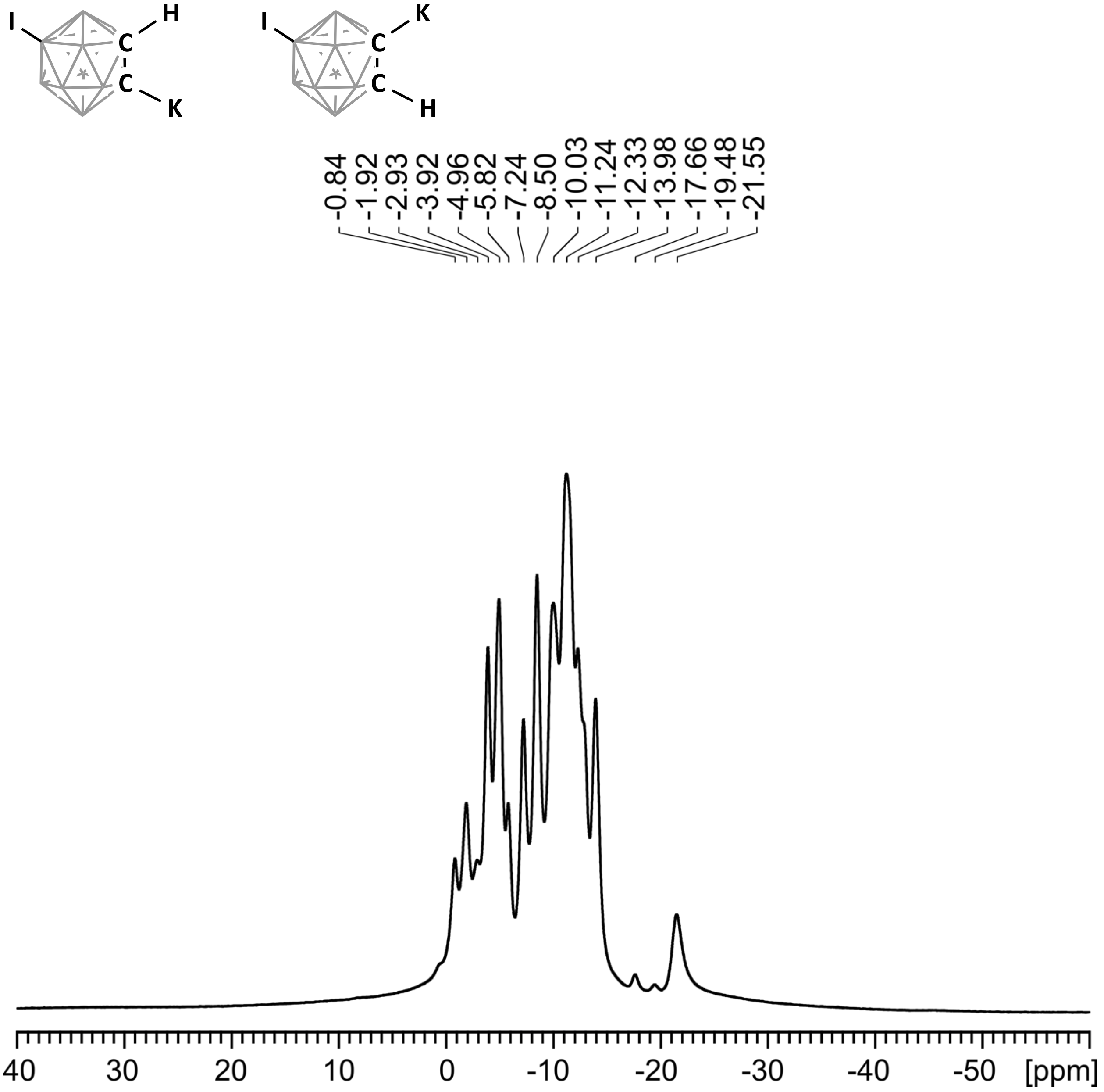

Figure S-8. The ^{11}B NMR spectrum of $[C_2B_{10}H_{10}I]K$ in THF at room temperature. Note that two isomers of the monodeprotonated cluster were observed.

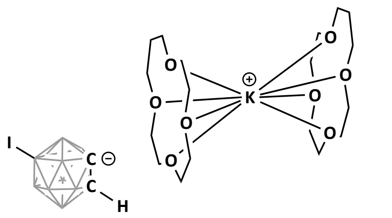

4

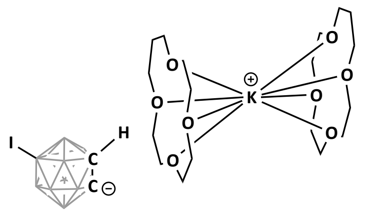

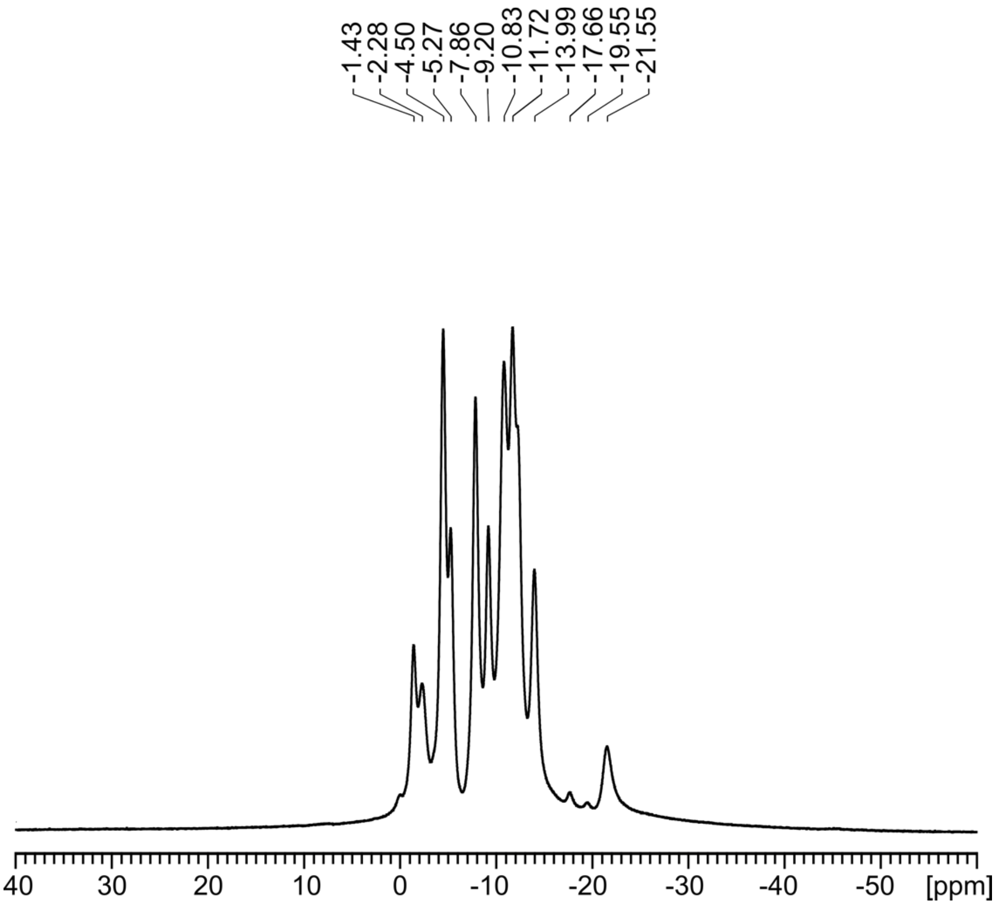

Figure S-9. The $^{11}B\{^1H\}$ NMR spectrum of $[K(12\text{-crown-}4)_2][C_2B_{10}H_{10}I]$ in THF at room temperature. Note that two isomers of the monodeprotonated cluster were observed.

$^{11}B\{^1H\}$ (THF): δ -1.43, -2.28, -4.50, -5.27, -7.86. -9.20, -10.83, -11.72, -13.99, -17.66, -19.55, -21.55

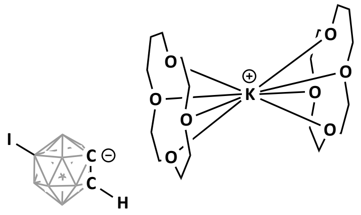

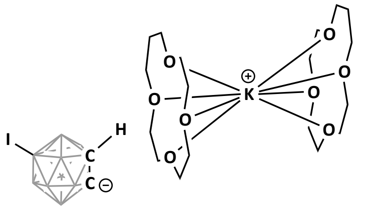

4

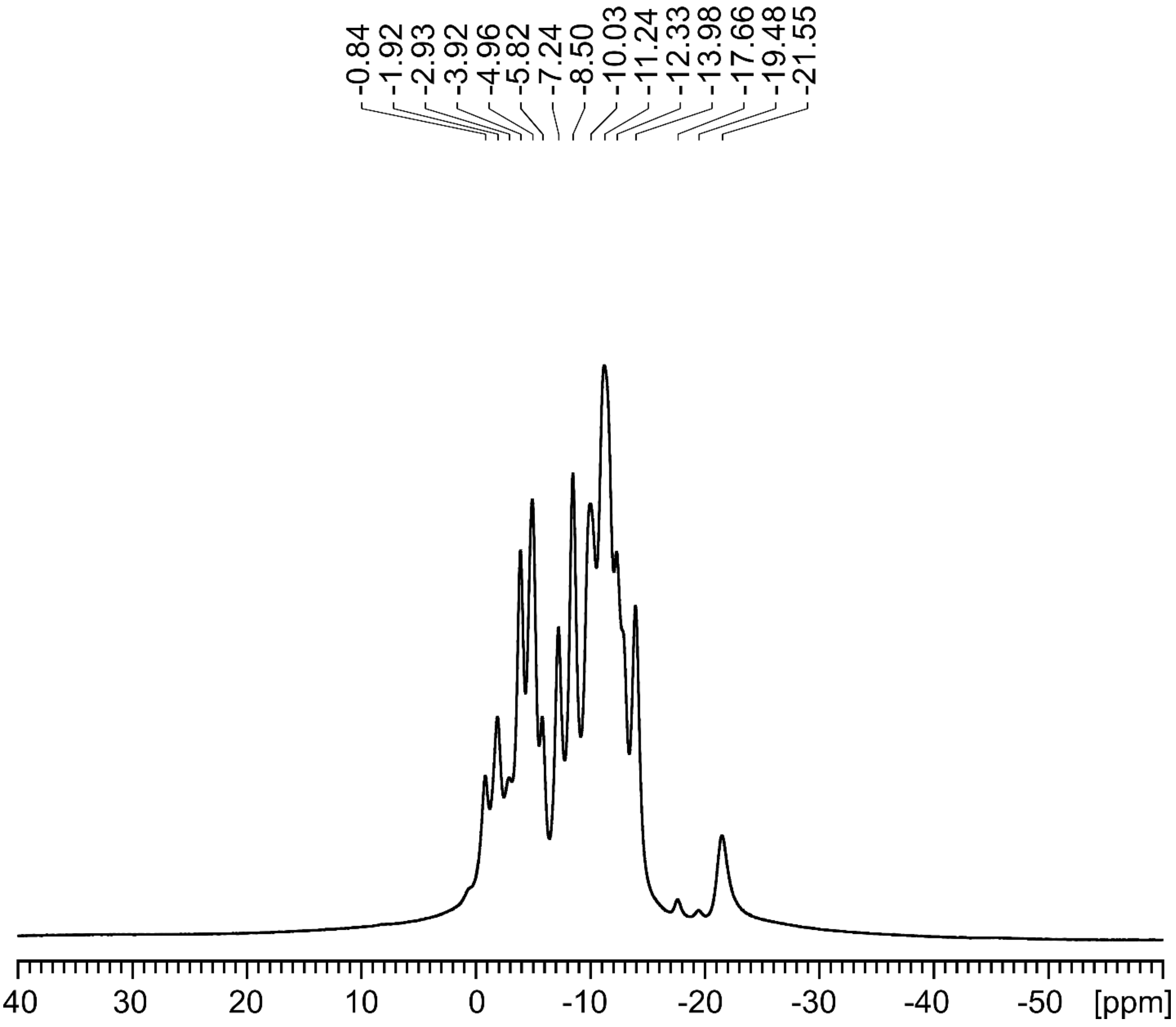

Figure S-10. The ^{11}B NMR spectrum of $[K(12\text{-crown-}4)_2][C_2B_{10}H_{10}I]$ in THF at room temperature. Note that two isomers of the monodeprotonated cluster were observed.

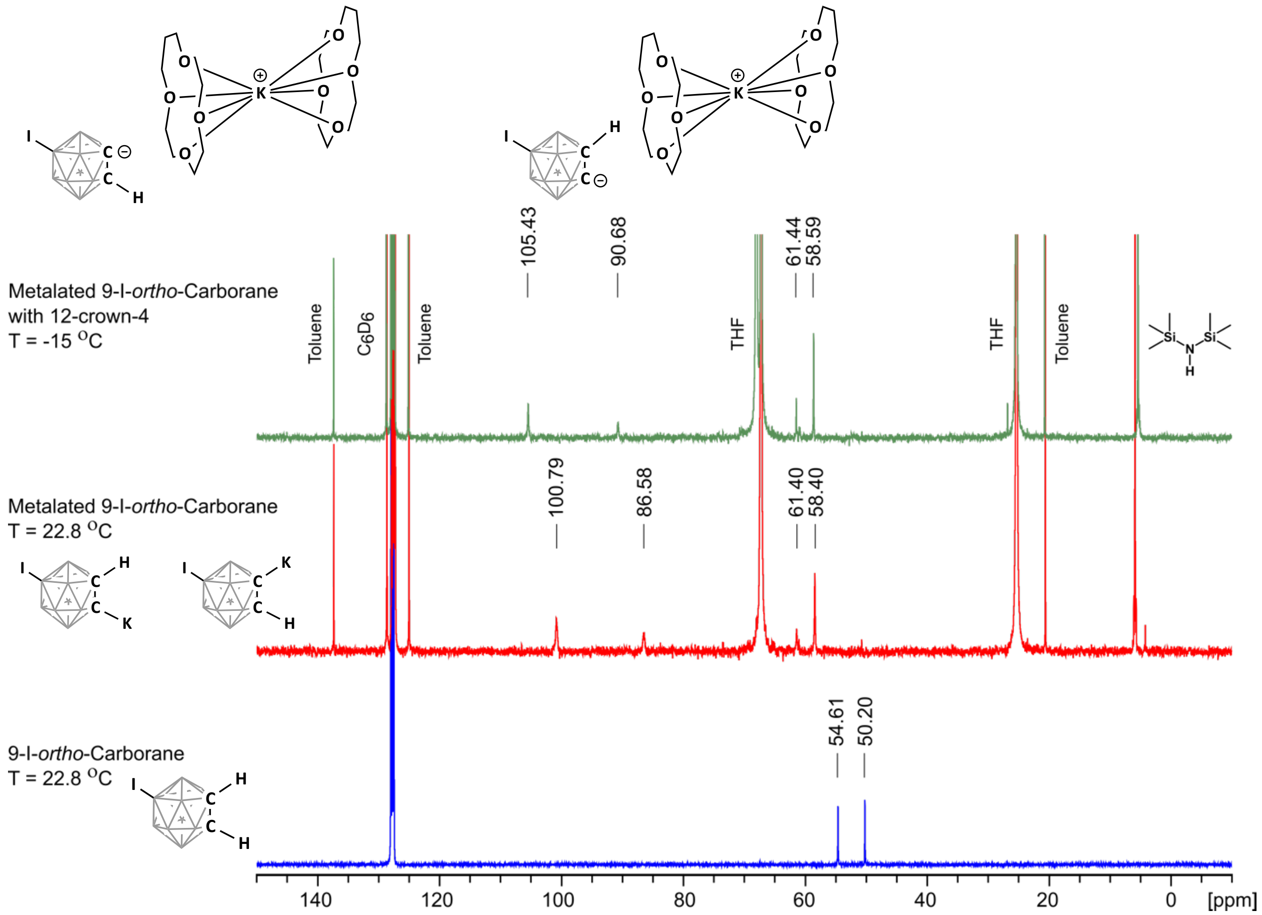

Figure S-11. The ^{13}C NMR spectra of $[K(12\text{-crown-}4)_2][C_2B_{10}H_{10}I]$ in THF before (middle) and after (top) addition of crown ether. The ^{13}C NMR spectrum of the starting protonated $C_2B_{10}H_{11}I$ is shown on the bottom for comparison.

For the completely separated ion pair after the addition of crown ether (top spectrum), signals at 58.6 ppm (major isomer) and 61.4 ppm (minor isomer) correspond to the protonated carbon atoms *C*(H). Signals at 105.4 ppm (major isomer) and 90.7 (minor isomer) correspond to the deprotonated "naked" carbanion atom.

For the metalated iodocarborane before addition of crown ether (spectrum in the middle), signals at 58.4 ppm (major isomer) and 61.4 ppm (minor isomer) correspond to the protonated carbon atoms *C*(H). Signals at 100.8 ppm (major isomer) and 86.6 (minor isomer) correspond to metalated carbon atoms *C*(K).

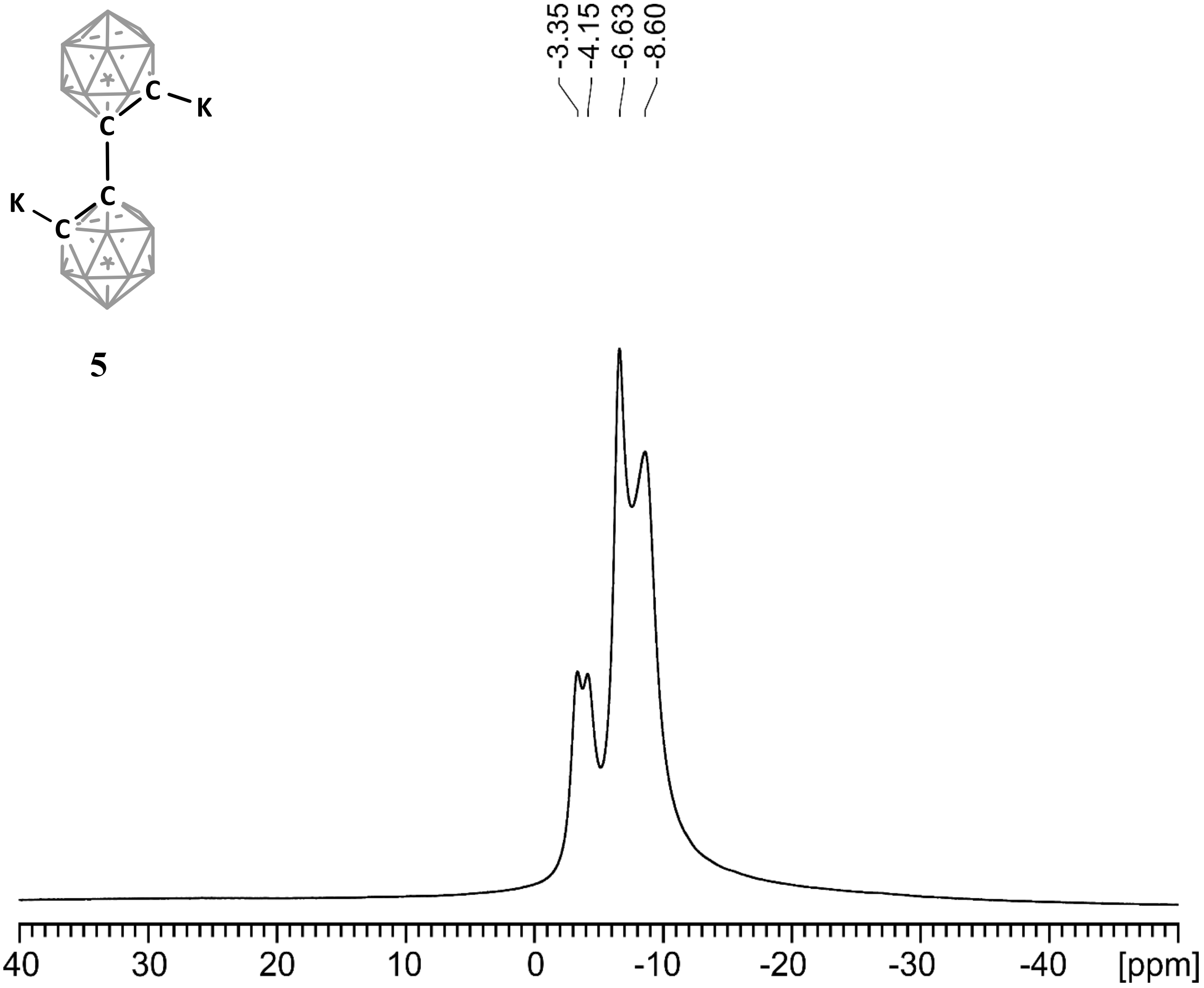

Figure S-12. The $^{11}B\{^1H\}$ NMR spectrum of $K_2(C_2B_{10}H_{10})_2$ in THF at room temperature.

$^{11}B\{^1H\}$ (THF): δ –3.3, –4.1, –6.6, –8.6.

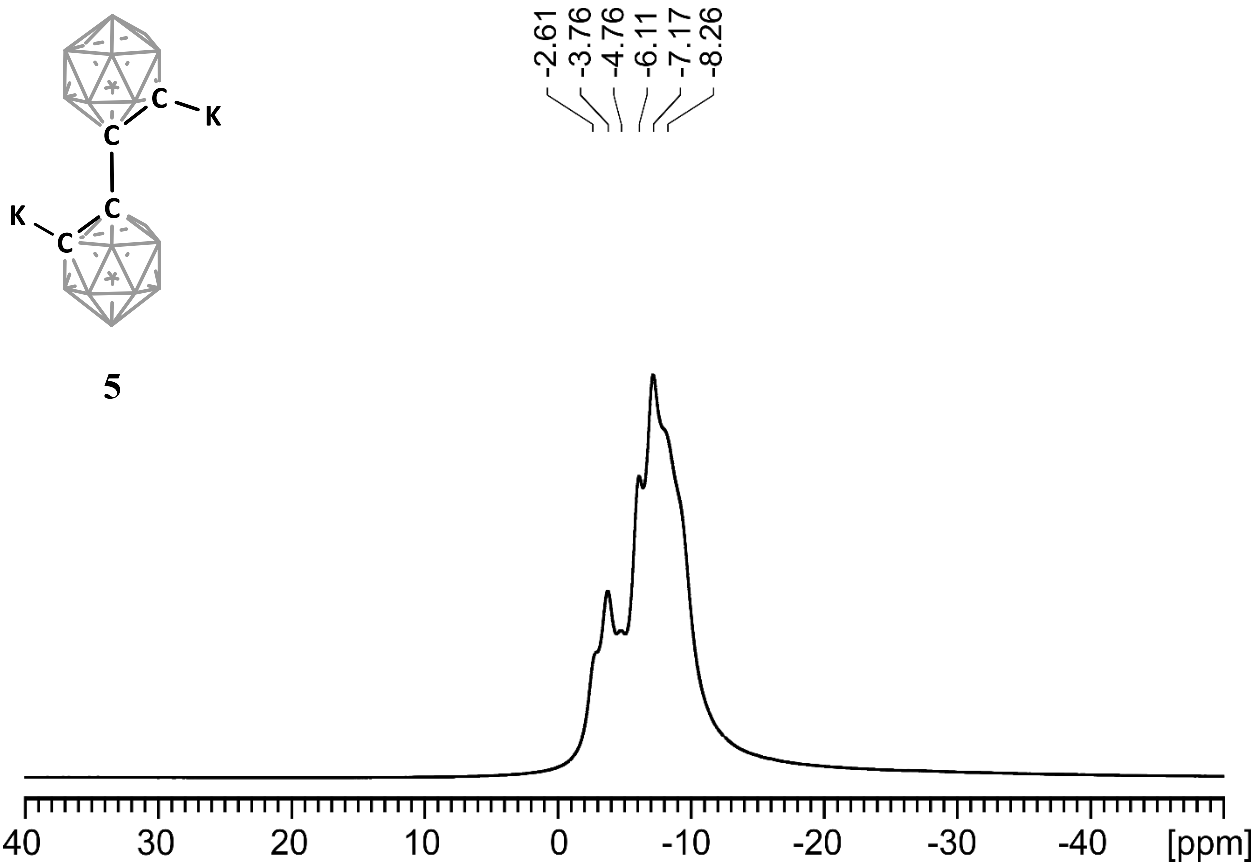

Figure S-13. The ^{11}B NMR spectrum of $K_2(C_2B_{10}H_{10})_2$ in THF at room temperature.

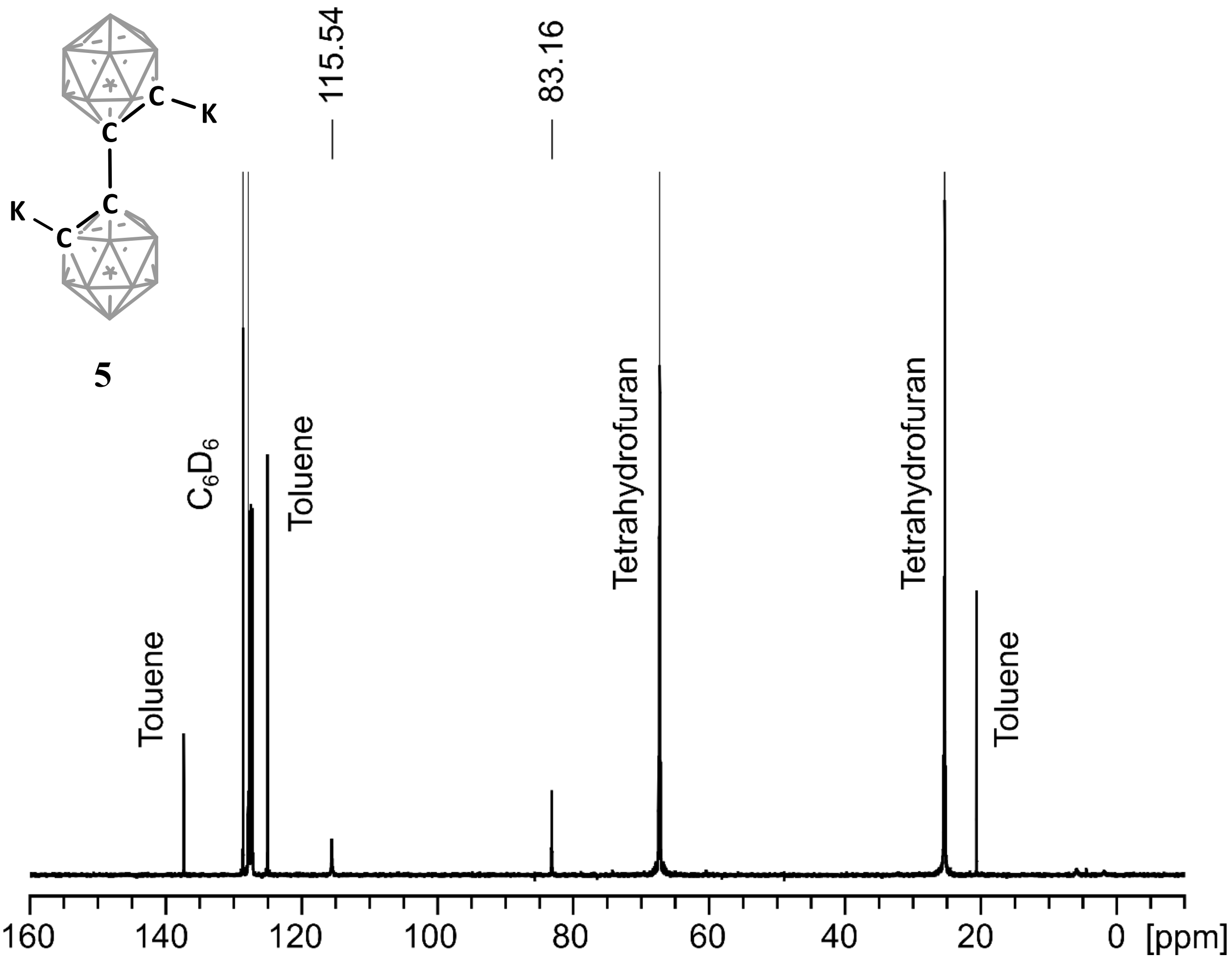

5

Figure S-14. The ^{13}C NMR spectrum of $K_2(C_2B_{10}H_{10})_2$ in THF at room temperature.

^{13}C (THF): δ 115.5 (*C*–H), 83.2 (*C*–*C*).

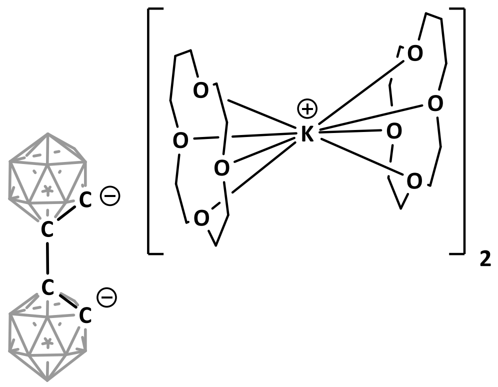

6

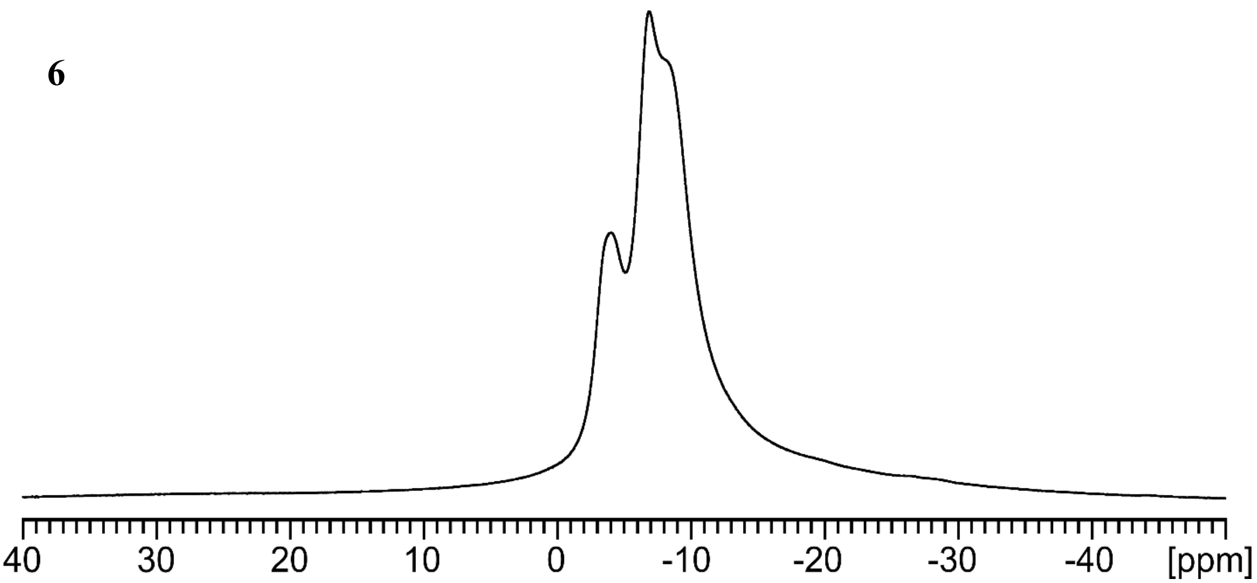

Figure S-15. The $^{11}B\{^1H\}$ NMR spectrum of $[K(12\text{-crown-}4)_2]_2[(C_2B_{10}H_{10})_2]$ in THF at room temperature.

$^{11}B\{^1H\}$ (THF): δ –4.1,–6.9, –8.4.

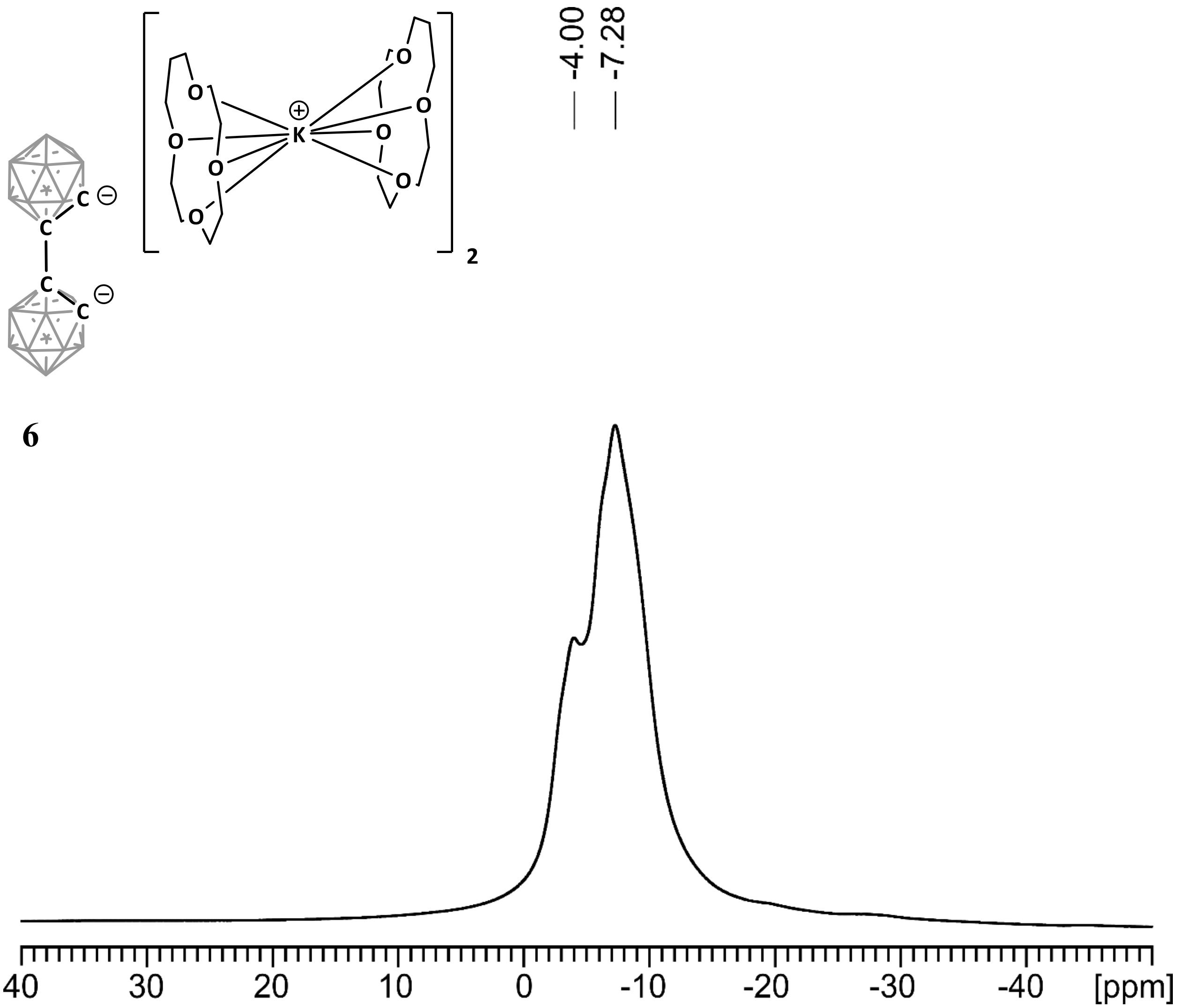

6

Figure S-16. The ^{11}B NMR spectrum of $[K(12\text{-crown-}4)_2]_2[(C_2B_{10}H_{10})_2]$ in THF at room temperature.

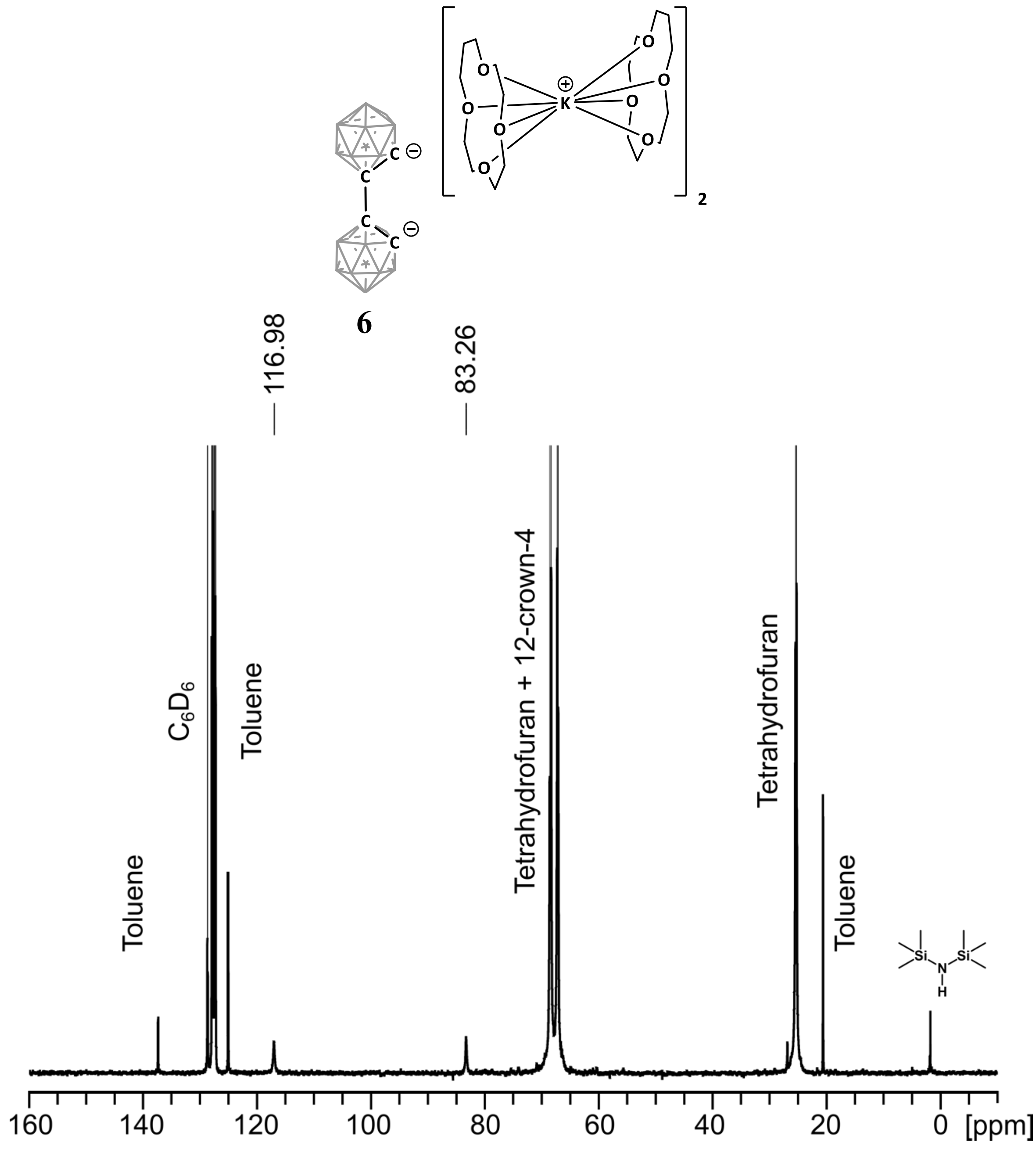

Figure S-17. The ^{13}C NMR spectrum of $[K(12\text{-crown-}4)_2]_2[(C_2B_{10}H_{10})_2]$ in THF at room temperature.

[13]C (THF): δ 117.0 ($C^{\ominus}$), 83.3 (*C*–*C*).

X-Ray Structure Determination, [K(18-crown-6)][$C_2B_{10}H_{11}$] (2) (md7095)

X-ray intensity data from pale yellow plate were collected at 100(2) K using a Bruker D8 QUEST diffractometer equipped with a PHOTON-100 CMOS area detector and an Incoatec microfocus source (Mo Kα radiation, λ = 0.71073 Å). The raw area detector data frames were reduced and corrected for absorption effects using the Bruker APEX3, SAINT+ and SADABS programs.[1,2] The structure was solved with SHELXT.[3] Subsequent difference Fourier calculations and full-matrix least-squares refinement against F^2 were performed with SHELXL-2018[3] using OLEX2.[4]

The compound crystallizes in the trigonal (rhombohedral) system. The pattern of systematic absences in the intensity data ruled out a *c*-glide symmetry element. Though disorder precludes unambiguous space group identification, the best solution was found in the acentric group *R*3*m*. The asymmetric unit in *R*3*m* consists of consists of a K atom, two partially occupied fragments of a disordered 12-crown-6 ether ligand, and four non-hydrogen atoms of an uncomplexed carborane cage. There is extensive disorder in the crystal, affecting the crown ether and the carborane cage. The potassium atom is located on a site with C_{3v} point symmetry. The crown ether is disordered about this site, and was modeled with two distinct, partially occupied components. The major component has an occupancy of 0.806(4) and consists of two oxygen atoms (O1, O2), both located on mirror planes, and two -CH_2- groups (C11, C12) on general crystallographic positions. The minor component (occupancy 0.194(4)) consists of one oxygen (O3) and two -CH_2- groups (C21, C22), all on general positions. For refinement, all ether C-O and C-C distances were restrained to be similar (SHELX SADI). The carborane cage has crystallographic C_{3v} point symmetry and is generated from four independent non-H atoms (X1-X4) per asymmetric unit. X-X bond distances were not useful in determining the carbon atom locations, as all X-X distances are similar. The carbon atoms were assigned to site X1 on the basis of atomic site occupation factors. Refinement of the X1-X4 site occupancies gave X1 = 1.13(5), X2 = 1.03(3), X3 = 1.03(3), X4 = 0.94(3). Site X1 was therefore the most reasonable candidate for the carbon site, and was refined as a mixed, disordered C/B site (C1/B1). Sites X2-X4 were refined as 100% boron (B2-B4). The C_{3v} point symmetry of the cage generates three total symmetry-equivalent C1/B1 sites. The C1/B1 site occupancy was therefore fixed at 2/3 C and 1/3 B to reflect the two total C sites per cage. This resulted in reasonable displacement parameters for this site, providing some support for the C atom assignment. All non-hydrogen atoms were refined with anisotropic displacement parameters. Crown ether hydrogen atoms were placed in geometrically idealized positions and included as riding atoms with *d*(C-H) = 0.99 Å and *U*iso(H) = 1.2*U*eq(C). All four carborane cage hydrogen atoms were located in Fourier difference maps. Those bonded to boron atoms B2-B4 were refined freely with B2 and B3 sharing a common isotropic displacement parameter. To maintain charge balance, the carborane cage must be a monoanion and must therefore be singly deprotonated. The deprotonation site is also disordered and could not be directly established, but it is assumed to be disordered over one of the B1/C1 sites. For refinement this hydrogen (H1) was refined with 2/3 occupancy, reflecting one absent H per three possible C sites. This treatment produces the required electroneutral composition of [Na(crown)$^+$][$C_2H_{11}B_{10}^-$], and reasonable H atom displacement parameters. The largest residual electron density peak in the final difference map is 0.26 $e^-/Å^3$, located 1.04 Å from C1/B1. A refined model in the higher symmetry space group *R*-3m also gave acceptable statistics (*R*1 = 0.053) but produced a centrosymmetric carborane cage, imposing further B/C disorder that is resolved in *R*3*m*. A model in space group *R*3 yielded similar results as in *R*3*m* but did not further resolve any disorder, and

used more refined parameters. The crown ether disorder was observed in all space groups tried. The absolute structure parameters at convergence was 0.5(2), and the crystal was therefore refined as an inversion twin.

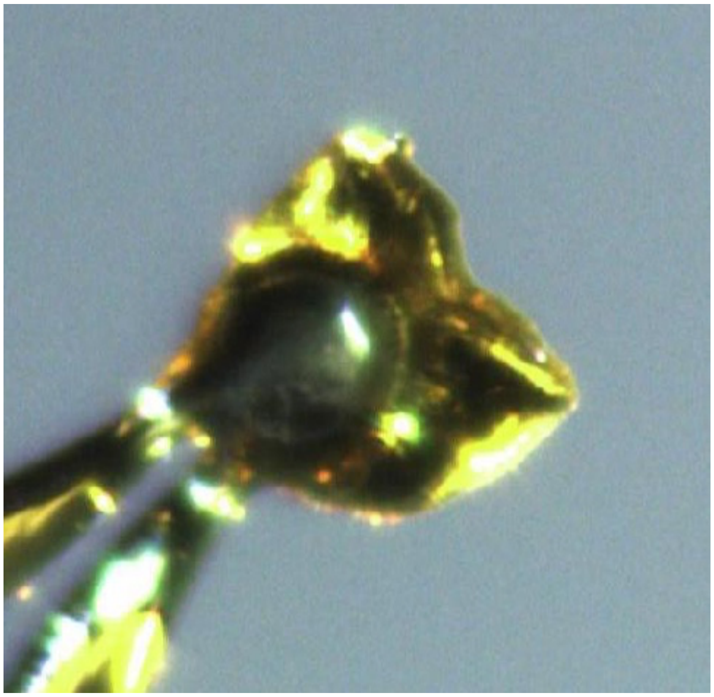

Figure S-18. Image of the data crystal.

(1) **APEX3** Version 2018.1-0 and **SAINT+** Version 8.38A. Bruker AXS, Inc., Madison, Wisconsin, USA, 2016.

(2) **SADABS**-2016/2: Krause, L., Herbst-Irmer, R., Sheldrick G.M. and Stalke D. *J. Appl. Cryst.* **2015**, *48*, 3-10.

(3) (a) **SHELXT**: Sheldrick, G.M. *Acta Cryst*. **2015**, *A71*, 3-8. (b) **SHELXL:** Sheldrick, G.M. *Acta Cryst*. **2015**, *C71*, 3-8.

(4) **OLEX2**: a complete structure solution, refinement and analysis program. Dolomanov, O. V., Bourhis, L. J., Gildea, R. J., Howard J. A. K. and Puschmann, H. *J. Appl. Cryst.* **2009**, *42*, 339-341.

Table S-1. Crystal data for [K(18-crown-6)][$C_2B_{10}H_{11}$] (2)

Identification code	md7095
Empirical formula	$C_{14}H_{35}B_{10}KO_6$
Formula weight	446.61
Temperature/K	100(2)
Crystal system	trigonal
Space group	R3m
a/Å	14.6250(9)
b/Å	14.6250(9)
c/Å	9.4818(7)
α/°	90
β/°	90
γ/°	120
Volume/Å^3	1756.4(3)
Z	3.00006
ρ_{calc}g/cm^3	1.267
μ/mm^{-1}	0.255
F(000)	708.0
Crystal size/mm^3	0.24 × 0.18 × 0.06
Radiation	MoKα (λ = 0.71073)
2Θ range for data collection/°	5.366 to 54.884
Index ranges	-18 ≤ h ≤ 18, -18 ≤ k ≤ 18, -12 ≤ l ≤ 12
Reflections collected	8456
Independent reflections	1004 [R_{int} = 0.0353, R_{sigma} = 0.0183]
Data/restraints/parameters	1004/10/80
Goodness-of-fit on F^2	1.083
Final R indexes [I>=2σ (I)]	R_1 = 0.0466, wR_2 = 0.1151
Final R indexes [all data]	R_1 = 0.0561, wR_2 = 0.1243
Largest diff. peak/hole / e Å^{-3}	0.26/-0.18
Flack parameter	0.5(2)

X-Ray Structure Determination, [K(18-crown-6)(THF)][$C_2B_{10}H_{10}I$] (3) **(CJ-103)**

X-ray intensity data from a colorless domed plate were collected at 100(2) K using a Bruker D8 QUEST diffractometer equipped with a PHOTON-100 CMOS area detector and an Incoatec microfocus source (Mo Kα radiation, λ = 0.71073 Å). The raw area detector data frames were reduced, scaled and corrected for absorption effects using the Bruker APEX3, SAINT+ and SADABS programs.[1,2] Final unit cell parameters were determined by least-squares refinement of 9322 reflections taken from the data set. The structure was solved with SHELXT.[3] Subsequent difference Fourier calculations and full-matrix least-squares refinement against F^2 were performed with SHELXL-2018[3] using OLEX2.[4]

The compound crystallizes in the triclinic system. The space group *P*-1 (No. 2) was confirmed by structure solution. The asymmetric unit consists of one complex. All non-hydrogen atoms were refined with anisotropic displacement parameters. Hydrogen atoms of the crown ether and THF ligands were located in Fourier difference maps before being placed in geometrically idealized positions and included as riding atoms with *d*(C-H) = 0.99 Å and *U*iso(H) = 1.2*U*eq(C). Hydrogen atoms bonded to boron and carbon atoms of the carborane cage were located and refined freely. The largest residual electron density peak in the final difference map is 1.20 e^-/Å^3, located 0.79 Å from O24. This likely arises from a minor crown ether disorder component, which was not modeled because of its small occupancy.

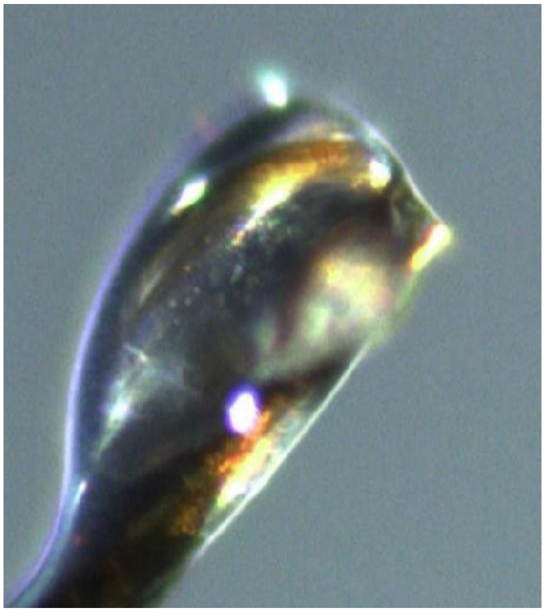

Figure S-19. Image of the data crystal

(1) **APEX3** Version 2016.5-0 and **SAINT+** Version 8.37A. Bruker AXS, Inc., Madison, Wisconsin, USA, 2016.

(2) **SADABS**-2016/2: Krause, L., Herbst-Irmer, R., Sheldrick G.M. and Stalke D. *J. Appl. Cryst.* **2015**, *48*, 3-10.

(3) (a) **SHELXT**: Sheldrick, G.M. *Acta Cryst*. **2015**, *A71*, 3-8. (b) **SHELXL:** Sheldrick, G.M. *Acta Cryst*. **2015**, *C71*, 3-8.

(4) **OLEX2**: a complete structure solution, refinement and analysis program. Dolomanov, O. V., Bourhis, L. J., Gildea, R. J., Howard J. A. K. and Puschmann, H. *J. Appl. Cryst.* **2009**, *42*, 339-341.

Table S-2. Crystal data for [K(18-crown-6)(THF)][$C_2B_{10}H_{10}I$] (3)

Identification code	cj103
Empirical formula	$C_{18}H_{42}B_{10}IKO_7$
Formula weight	644.61
Temperature/K	100(2)
Crystal system	triclinic
Space group	P-1
a/Å	9.0729(6)
b/Å	12.0904(7)
c/Å	14.4358(9)
α/°	78.659(2)
β/°	85.435(2)
γ/°	79.339(2)
Volume/Å^3	1524.28(16)
Z	2
ρ_{calc}g/cm^3	1.404
μ/mm^{-1}	1.221
F(000)	656.0
Crystal size/mm^3	0.4 × 0.34 × 0.16
Radiation	MoKα (λ = 0.71073)
2Θ range for data collection/°	4.09 to 71.37
Index ranges	-14 ≤ h ≤ 14, -19 ≤ k ≤ 19, -23 ≤ l ≤ 23
Reflections collected	84229
Independent reflections	13950 [R_{int} = 0.0310, R_{sigma} = 0.0178]
Data/restraints/parameters	13950/0/375
Goodness-of-fit on F^2	1.080
Final R indexes [I>=2σ (I)]	R_1 = 0.0235, wR_2 = 0.0609
Final R indexes [all data]	R_1 = 0.0257, wR_2 = 0.0622
Largest diff. peak/hole / e Å^{-3}	1.20/-0.53

X-Ray Structure Determination, [K(12-crown-4)$_2$][$C_2B_{10}H_{10}I$] (4) (CJ-117)

X-ray intensity data from a colorless block crystal were collected at 100(2) K using a Bruker D8 QUEST diffractometer equipped with a PHOTON-100 CMOS area detector and an Incoatec microfocus source (Mo Kα radiation, λ = 0.71073 Å). The crystals decompose rapidly when removed from the mother liquor and must be transferred to the cold stream within seconds. The raw area detector data frames were reduced, scaled and corrected for absorption effects using the Bruker APEX3, SAINT+ and SADABS programs.[1,2] The structure was solved with SHELXT.[3] Subsequent difference Fourier calculations and full-matrix least-squares refinement against F^2 were performed with SHELXL-2018[3] using OLEX2.[4]

The compound crystallizes in the monoclinic system. The pattern of systematic absences in the intensity data was consistent with the space groups $P2_1$ and $P2_1/m$; intensity statistics suggested an acentric structure. The solution program XT found $P2_1$, which was confirmed by obtaining a reasonable and stable solution and refinement. The space group was further checked with ADDSYM, which found no higher symmetry.[5] The asymmetric unit consists of two crystallographically independent but chemically identical $[K(C_8H_{16}O_4)_2]^+$ cations and two $[C_2B_{10}H_{10}I]^-$ anions. Boron and carbon sites in the carborane cage were assigned as follows: The position of one of the two carborane C atoms is fixed by synthesis, opposite to the iodine atom (C2 and C12). The position of the second carborane carbon (C1 and C11) in each cage was assigned by evaluating the bond distances to the surrounding five atoms and the displacement parameters of the candidate sites. There are five potential sites. For both independent carborane anions, bond distances from the second C site to the surrounding sites were on average shorter than those of the other candidate sites (*ca.* 1.70 vs. 1.75 Å). Secondly, if all five sites were assigned as boron, the displacement parameter of the C site was significantly smaller compared to the surrounding sites, indicating greater electron density at that cage site than contributed by a boron atom. These two observations are consistent with a carbon atom located at sites C1 and C11. The bond distance and displacement parameter observations also held for the C2 and C12 sites. Both 12-crown-4 ligands of cation K2 are disordered and were modeled with two orientations each (O41-O44/C41-C48 A/B and O51-O54/C51-C58 A/B). Major populations for each ligand refined to: O41A-C48A = 0.792(5) and O51A-C58A = 0.612(9). 1,2- and 1,3- distances within the disordered ligands were restrained to be similar to those in the ordered ligand O21-C28 using SHELX SAME instructions. All non-hydrogen atoms were refined with anisotropic displacement parameters. Hydrogen atoms of the crown ether ligands were placed in geometrically idealized positions and included as riding atoms with *d*(C-H) = 0.99 Å and Uiso(H) = 1.2Ueq(C). Reasonable positions for the carborane cage hydrogen atoms were located in difference Fourier maps. All but two (H1(B1) and H12(B12)) could be refined freely. H1 was fixed at *d*(B-H) = 1.12 Å and both H1 and H12 were refined with Uiso(H) = 1.2Ueq(B). No electron density was observed in the vicinity of C1 and C11, calculated down to the 0.25 e^-/A^3 level. These two carbon atoms are thus deprotonated sites. The largest residual electron density peak in the final difference map is 1.52 $e^-/Å^3$, located 1.50 Å from H1. This peak is not chemically sensible, and likely is the iodine atom contribution of a very small disorder fraction of carborane anion I1. Near the final refinement cycles, the absolute structure (Flack) parameter was 0.16(2), indicating the data crystal possessed a minor twin component. An inversion twinning matrix was included in the final cycles, with the Flack parameter as the minor twin volume fraction.

(1) **APEX3** Version 2016.5-0 and **SAINT+** Version 8.37A. Bruker AXS, Inc., Madison, Wisconsin, USA, 2016.

(2) **SADABS**-2016/2: Krause, L., Herbst-Irmer, R., Sheldrick G.M. and Stalke D. *J. Appl. Cryst.* **2015**, *48*, 3-10.

(3) (a) **SHELXT**: Sheldrick, G.M. *Acta Cryst*. **2015**, *A71*, 3-8. (b) **SHELXL:** Sheldrick, G.M. *Acta Cryst*. **2015**, *C71*, 3-8.

(4) **OLEX2**: a complete structure solution, refinement and analysis program. Dolomanov, O. V., Bourhis, L. J., Gildea, R. J., Howard J. A. K. and Puschmann, H. *J. Appl. Cryst.* **2009**, *42*, 339-341.

(5) **ADDSYM**: (a) LePage, Y. *J. Appl. Crystallogr.* **1987**, *20*, 264-269. (b) Spek, A. L. *J. Appl. Crystallogr.*, **1988**, *21*, 578-579. (c) Spek, A. L. *Acta Crystallogr., Sect A* **1990**, *46*, C34. (d) **PLATON**: Spek, A.L. *Acta Cryst.,* **2009**, *D65*, 148-155.

Table S-3. Crystal data for $[K(12\text{-crown-}4)_2][C_2B_{10}H_{10}I]$ (4)

Identification code	cj117
Empirical formula	$C_{18}H_{42}B_{10}IKO_8$
Formula weight	660.61
Temperature/K	100(2)
Crystal system	monoclinic
Space group	$P2_1$
a/Å	12.2342(4)
b/Å	15.5074(5)
c/Å	16.1469(5)
α/°	90
β/°	92.250(2)
γ/°	90
Volume/Å^3	3061.04(17)
Z	4
ρ_{calc}g/cm^3	1.433
μ/mm^{-1}	1.220
F(000)	1344.0
Crystal size/mm^3	0.44 × 0.4 × 0.2
Radiation	MoKα (λ = 0.71073)
2Θ range for data collection/°	2.524 to 60.128
Index ranges	$-17 \le h \le 17$, $-21 \le k \le 21$, $-22 \le l \le 22$
Reflections collected	144478
Independent reflections	17911 [R_{int} = 0.0421, R_{sigma} = 0.0248]
Data/restraints/parameters	17911/314/925
Goodness-of-fit on F^2	1.025
Final R indexes [I>=2σ (I)]	R_1 = 0.0398, wR_2 = 0.1031
Final R indexes [all data]	R_1 = 0.0422, wR_2 = 0.1049
Largest diff. peak/hole / e Å^{-3}	1.52/-1.11
Flack parameter	0.163(17)

X-Ray Structure Determination, $[K(THF)_2]_2(C_2B_{10}H_{10})_2$ (5) (CJ-89)

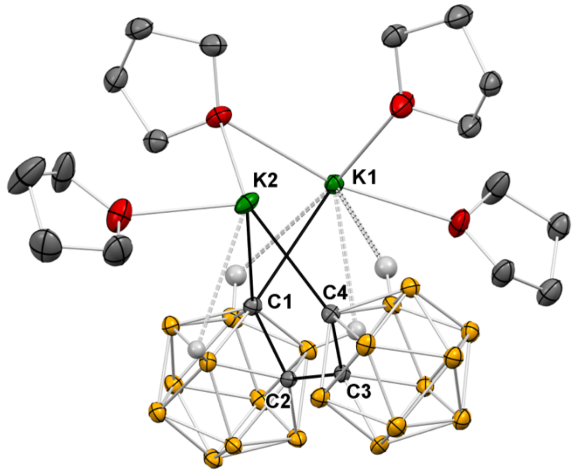

Figure S-20. Displacement ellipsoid plot (50% probability) of $[K(THF)_2]_2(C_2B_{10}H_{10})_2$ (**5**). Hydrogen atoms on boron atoms of the cluster and on carbon atoms of the THF ligand have been omitted for clarity except for bridging B−H···K interactions. Selected interatomic distances (Å): K1−C1 = 3.175(1), K2–C1 2.997(1), K2–C4 2.843(1).

X-ray intensity data from a colorless block were collected at 100(2) K using a Bruker D8 QUEST diffractometer equipped with a PHOTON-100 CMOS area detector and an Incoatec microfocus source (Mo Kα radiation, λ = 0.71073 Å). The raw area detector data frames were reduced and corrected for absorption effects using the Bruker APEX3, SAINT+ and SADABS programs.[1,2] The structure was solved with SHELXT.[3] Subsequent difference Fourier calculations and full-matrix least-squares refinement against F^2 were performed with SHELXL-2018[3] using OLEX2.[4]

The compound crystallizes in the monoclinic system. The pattern of systematic absences in the intensity data was consistent with the space group $P2_1/n$, which was confirmed by structure solution. The asymmetric unit consists of two crystallographically unique potassium atoms, one carborane species and four THF molecules. One THF (O4, C41-C44) is disordered over two closely spaced orientations A/B with a major population of 0.657(8). 1,2 and 1,3 bond distances of both disorder components were restrained to be similar to those of ordered THF O1/C11-C14 with SHELX SAME instructions. All non-hydrogen atoms were refined with anisotropic displacement parameters. Those of the disordered THF atoms were restrained to physically reasonable forms using SHELX RIGU instructions. Hydrogen atoms were in general located in difference Fourier maps. THF hydrogen atoms were placed in geometrically idealized positions and included as riding atoms with d(C-H) = 0.99 Å and Uiso(H) = 1.2Ueq(C). Hydrogen atoms of the carborane unit were refined freely. The largest residual electron density peak in the final difference map is 0.49 e^-/Å^3, located 0.69 Å from H44D.

(1) **APEX3** Version 2018.1-0 and **SAINT+** Version 8.38A. Bruker AXS, Inc., Madison, Wisconsin, USA, 2016.

(2) **SADABS**-2016/2: Krause, L., Herbst-Irmer, R., Sheldrick G.M. and Stalke D. *J. Appl. Cryst.* **2015**, *48*, 3-10.

(3) (a) **SHELXT**: Sheldrick, G.M. *Acta Cryst*. **2015**, *A71*, 3-8. (b) **SHELXL:** Sheldrick, G.M. *Acta Cryst*. **2015**, *C71*, 3-8.

(4) **OLEX2**: a complete structure solution, refinement and analysis program. Dolomanov, O. V., Bourhis, L. J., Gildea, R. J., Howard J. A. K. and Puschmann, H. *J. Appl. Cryst.* **2009**, *42*, 339-341.

Table S-4. Crystal data for $[K(THF)_2]_2(C_2B_{10}H_{10})_2$ (5)

Identification code	cj89
Empirical formula	$C_{20}H_{52}B_{20}K_2O_4$
Formula weight	651.01
Temperature/K	100(2)
Crystal system	monoclinic
Space group	$P2_1/n$
a/Å	14.4831(7)
b/Å	11.9030(6)
c/Å	21.6219(11)
α/°	90
β/°	108.566(2)
γ/°	90
Volume/Å^3	3533.5(3)
Z	4
ρ_{calc}g/cm^3	1.224
μ/mm^{-1}	0.297
F(000)	1368.0
Crystal size/mm^3	0.44 × 0.28 × 0.24
Radiation	MoKα (λ = 0.71073)
2Θ range for data collection/°	3 to 55.184
Index ranges	$-18 \le h \le 18$, $-15 \le k \le 15$, $-28 \le l \le 28$
Reflections collected	63589
Independent reflections	8155 [R_{int} = 0.0621, R_{sigma} = 0.0312]
Data/restraints/parameters	8155/90/527
Goodness-of-fit on F^2	1.043
Final R indexes [I>=2σ (I)]	R_1 = 0.0361, wR_2 = 0.0868
Final R indexes [all data]	R_1 = 0.0474, wR_2 = 0.0924
Largest diff. peak/hole / e Å^{-3}	0.49/-0.42

X-Ray Structure Determination, $[K(12\text{-crown-}4)_2]_2(C_2B_{10}H_{10})_2\cdot 4(C_4H_8O)$ (6) (CJ-121)

X-ray intensity data from an irregular colorless block were collected at 100(2) K using a Bruker D8 QUEST diffractometer equipped with a PHOTON-100 CMOS area detector and an Incoatec microfocus source (Mo Kα radiation, λ = 0.71073 Å). The crystals decompose within several seconds outside the mother liquor, even under oil, and must be transferred as rapidly as possible to the cold stream. The raw area detector data frames were reduced, scaled and corrected for absorption effects using the Bruker APEX3, SAINT+ and SADABS programs.[1,2] The structure was solved with SHELXT.[3] Subsequent difference Fourier calculations and full-matrix least-squares refinement against F^2 were performed with SHELXL-2018[3] using OLEX2.[4]

The compound crystallizes in the triclinic system. The space group *P*-1 (No. 2) was confirmed by structure solution. The asymmetric unit consists of one $[K(C_8H_{16}O_4)_2]^+$ cation, half of one $[C_4H_{20}B_{20}]^{2-}$ dianion and two independent THF molecules. The $[C_4H_{20}B_{20}]^{2-}$ dianion is located on a crystallographic inversion center. One crown ether ligand of the cation (O21-O24/C21-C28) is disordered over two orientations with a major disorder population fraction of 0.913(1). The geometries of both disordered crown ether components were restrained to be similar to that of the ordered crown ether (O11-O14/C11-C18) using SAME instructions. Both THF molecules are disordered and were modeled with three components each. Total occupancies for each THF were constrained to sum to one, and *d*(C-O) = 1.44(2) Å and *d*(C-C) = 1.49(2) Å distance restraints were applied to maintain chemically reasonable THF geometries. All non-hydrogen atoms were refined with anisotropic displacement parameters except for the disordered THF molecules (isotropic). Hydrogen atoms bonded to carbon were located in Fourier difference maps before being placed in geometrically idealized positions and included as riding atoms with *d*(C-H) = 0.99 Å and Uiso(H) = 1.2Ueq(C). Hydrogen atoms bonded to boron were located and refined freely. B/C assignments in the unique half-cage were straightforward based on analysis of the displacement parameters and average distances to the five surrounding atoms. The difference map clearly confirmed the absence of a hydrogen atom near C1, as no electron density greater than the 0.22 $e^-/Å^3$ level was observed near C1. The largest residual electron density peak in the final difference map is 0.93 $e^-/Å^3$, located 0.90 Å from C28A.

(1) **APEX3** Version 2016.5-0 and **SAINT+** Version 8.37A. Bruker AXS, Inc., Madison, Wisconsin, USA, 2016.

(2) **SADABS**-2016/2: Krause, L., Herbst-Irmer, R., Sheldrick G.M. and Stalke D. *J. Appl. Cryst.* **2015**, *48*, 3-10.

(3) (a) **SHELXT**: Sheldrick, G.M. *Acta Cryst*. **2015**, *A71*, 3-8. (b) **SHELXL:** Sheldrick, G.M. *Acta Cryst*. **2015**, *C71*, 3-8.

(4) **OLEX2**: a complete structure solution, refinement and analysis program. Dolomanov, O. V., Bourhis, L. J., Gildea, R. J., Howard J. A. K. and Puschmann, H. *J. Appl. Cryst.* **2009**, *42*, 339-341.

Table S-5. Crystal data $[K(12\text{-crown-}4)_2]_2(C_2B_{10}H_{10})_2\cdot4(C_4H_8O)$ (6)

Identification code	cj121
Empirical formula	$C_{52}H_{116}B_{20}K_2O_{20}$
Formula weight	1355.84
Temperature/K	100(2)
Crystal system	triclinic
Space group	P-1
a/Å	11.3462(4)
b/Å	13.3115(4)
c/Å	13.4954(4)
α/°	103.5080(10)
β/°	109.0800(10)
γ/°	96.6940(10)
Volume/Å^3	1831.27(10)
Z	1
ρ_{calc}g/cm^3	1.229
μ/mm^{-1}	0.194
F(000)	726.0
Crystal size/mm^3	0.5 × 0.44 × 0.3
Radiation	MoKα (λ = 0.71073)
2Θ range for data collection/°	3.884 to 60.44
Index ranges	$-15 \le h \le 15$, $-18 \le k \le 18$, $-18 \le l \le 19$
Reflections collected	99767
Independent reflections	10684 [R_{int} = 0.0366, R_{sigma} = 0.0158]
Data/restraints/parameters	10684/133/536
Goodness-of-fit on F^2	1.036
Final R indexes [I>=2σ (I)]	R_1 = 0.0479, wR_2 = 0.1295
Final R indexes [all data]	R_1 = 0.0533, wR_2 = 0.1345
Largest diff. peak/hole / e Å^{-3}	0.93/-0.58

X-Ray Structure Determination, [K(18-crown-6)]$_2$($C_2B_{10}H_{10}$)$_2$·(C_7H_8)$_3$ (7) (CJ-105)

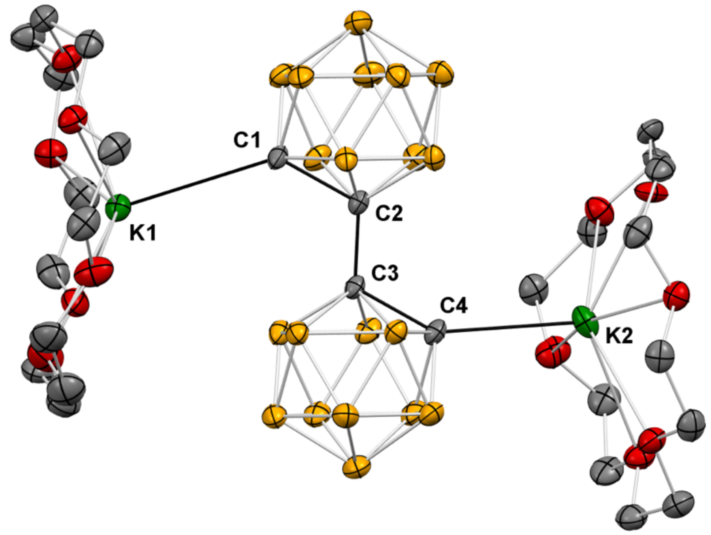

Figure S-21. Displacement ellipsoid plot (50% probability) of [K(18-crown-6)]$_2$($C_2B_{10}H_{10}$)$_2$. Hydrogen atoms on boron atoms of the cluster and on carbon atoms of the crown ether ligand ligand have been omitted for clarity. Toluene solvent molecules are not shown. Selected interatomic distances (Å): K1−C1 = 3.037(2), K2–C1 = 2.862(2).

X-ray intensity data from an irregular yellow chunk were collected at 100(2) K using a Bruker D8 QUEST diffractometer equipped with a PHOTON-100 CMOS area detector and an Incoatec microfocus source (Mo Kα radiation, λ = 0.71073 Å). The raw area detector data frames were reduced, scaled and corrected for absorption effects using the Bruker APEX3, SAINT+ and SADABS programs.[1,2] Final unit cell parameters were determined by least-squares refinement of 9638 reflections taken from the data set. The structure was solved with SHELXT.[3] Subsequent difference Fourier calculations and full-matrix least-squares refinement against F^2 were performed with SHELXL-2018[3] using OLEX2.[4]

The compound crystallizes in the monoclinic system. The pattern of systematic absences in the intensity data was consistent with the space group $P2_1/n$, which was confirmed by structure solution. The asymmetric unit consists of one [$K_2(C_{12}H_{24}O_6)_2(C_4B_{20}H_{20})$] complex and three crystallographically independent toluene molecules. One 18-crown-6-ether ligand (O21-O26, C23-C34) is disordered and was modeled with two components. The major disorder component occupancy refined to 0.804(2). 1,2- and 1,3- C-C and C-O distances in the minor component were restrained to be similar to those in the major using a SHELX SAME instruction. Two toluene molecules (C41-C47 and C51-C57) are disordered with two components each. Geometries of the minor components were restrained to be similar to those of the major using SAME instructions. An additional FLAT instruction was necessary to maintain planarity for C51B-C57B. Major toluene disorder component occupancies refined to C41A-C47A = 0.831(4) and C51A-C57A = 0.890(4). All non-hydrogen atoms were refined with anisotropic displacement parameters (*adp*s) except for the minor toluene disorder component atoms (isotropic). *Adp*s of nearly superimposed component atoms of the disordered crown ether were held equal. Hydrogen atoms bonded to carbon were located in Fourier difference maps before being placed in geometrically idealized positions and included as riding atoms with d(C-H) = 0.95 Å and Uiso(H) = 1.2Ueq(C) for arene

hydrogen atoms, *d*(C-H) = 0.99 Å and *U*iso(H) = 1.2*U*eq(C) for methylene hydrogen atoms, and *d*(C-H) = 0.98 Å and *U*iso(H) = 1.5*U*eq(C) for methyl hydrogens. The methyl hydrogens were allowed to rotate as a rigid group to the orientation of maximum observed electron density. Hydrogen atoms of the bis(carborane) cages were located and refined freely. The largest residual electron density peak in the final difference map is 1.07 $e^-/Å^3$, located 0.71 Å from K2.

(1) **APEX3** Version 2016.5-0 and **SAINT+** Version 8.37A. Bruker AXS, Inc., Madison, Wisconsin, USA, 2016.

(2) **SADABS**-2016/2: Krause, L., Herbst-Irmer, R., Sheldrick G.M. and Stalke D. *J. Appl. Cryst.* **2015**, *48*, 3-10.

(3) (a) **SHELXT**: Sheldrick, G.M. *Acta Cryst*. **2015**, *A71*, 3-8. (b) **SHELXL:** Sheldrick, G.M. *Acta Cryst*. **2015**, *C71*, 3-8.

(4) **OLEX2**: a complete structure solution, refinement and analysis program. Dolomanov, O. V., Bourhis, L. J., Gildea, R. J., Howard J. A. K. and Puschmann, H. *J. Appl. Cryst.* **2009**, *42*, 339-341.

Table S-6. Crystal data for $[K(18\text{-crown-}6)]_2(C_2B_{10}H_{10})_2\cdot(C_7H_8)_3$

Identification code	cj105
Empirical formula	$C_{49}H_{92}B_{20}K_2O_{12}$
Formula weight	1167.62
Temperature/K	100(2)
Crystal system	monoclinic
Space group	$P2_1/n$
a/Å	14.5139(6)
b/Å	19.6651(8)
c/Å	22.9425(10)
α/°	90
β/°	99.114(2)
γ/°	90
Volume/Å^3	6465.5(5)
Z	4
ρ_{calc}g/cm^3	1.200
μ/mm^{-1}	0.201
F(000)	2480.0
Crystal size/mm^3	0.6 × 0.48 × 0.38
Radiation	MoKα (λ = 0.71073)
2Θ range for data collection/°	2.742 to 55.132
Index ranges	$-18 \le h \le 18$, $-25 \le k \le 25$, $-29 \le l \le 29$
Reflections collected	164948
Independent reflections	14940 [R_{int} = 0.0735, R_{sigma} = 0.0322]
Data/restraints/parameters	14940/104/944
Goodness-of-fit on F^2	1.023
Final R indexes [I>=2σ (I)]	R_1 = 0.0526, wR_2 = 0.1280
Final R indexes [all data]	R_1 = 0.0738, wR_2 = 0.1411
Largest diff. peak/hole / e Å^{-3}	1.07/-0.75

Computational details.

The density functional theory calculations were carried out utilizing SCM ADF software package.[3] Molecular geometries were optimized using the hybrid PBE0 functional and the augmented by diffuse functions ATZP basis set with scalar relativistic effects considered using the ZORA formalism. Iodine atom was considered within the TZP basis set. Frequency calculations were carried out to confirm the local minimum results. Solvent effects (THF) were considered within COSMO model.

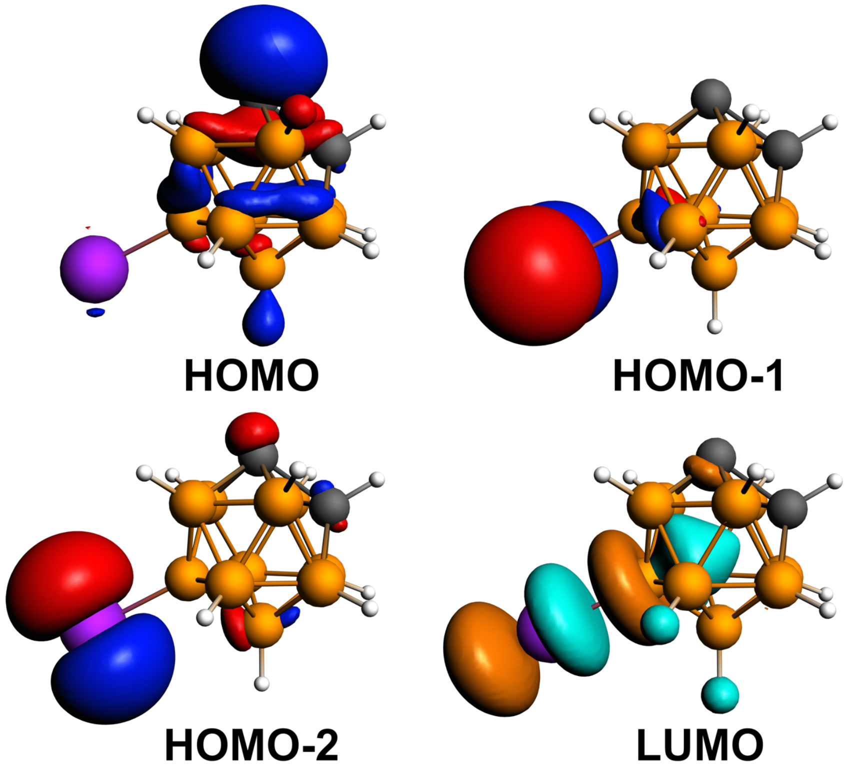

Figure S-22. HOMO, HOMO-1, HOMO-2, and LUMO for $[C_2B_{10}H_{10}I]^-$ (isosurface = 0.035).

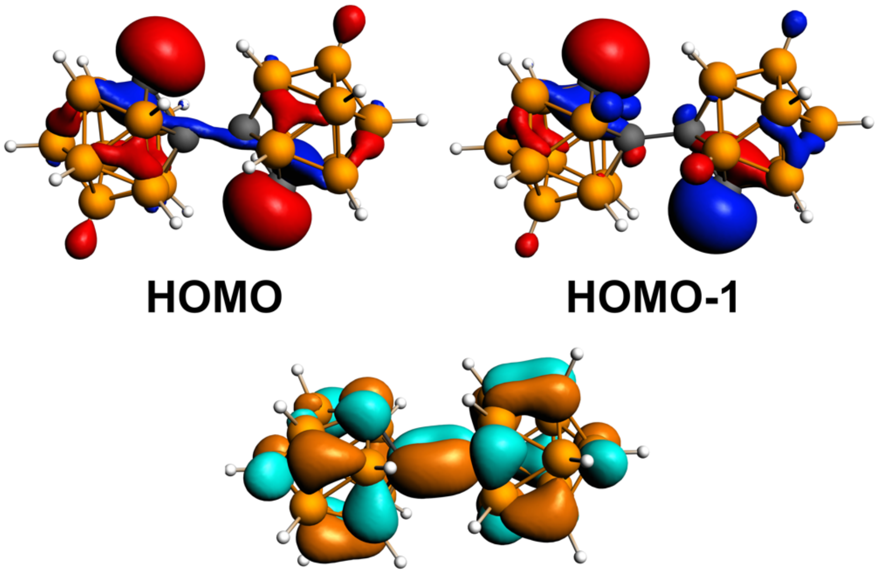

Figure S-23. HOMO, HOMO-1, and LUMO for $[(C_2B_{10}H_{10})_2]^{2-}$ (isosurface = 0.035).

Table S-7. The optimized geometry coordinates for $[C_2B_{10}H_{10}I]^{-}$

Atom	X	Y	Z (Angstrom)
1.H	-2.596575	1.522282	6.218885
2.B	-3.105922	3.610188	7.647591
3.H	-2.892138	4.413048	6.799752
4.B	-3.533278	3.048147	10.379331
5.H	-3.688000	3.523220	11.459005
6.B	-3.349018	1.303796	10.084320
7.H	-3.413788	0.509251	10.967840
8.B	-3.966171	0.974912	8.438298
9.H	-4.468346	-0.064315	8.145539
10.B	-4.517174	2.526773	7.741061
11.C	-1.863285	2.697875	8.289128

12.C	-2.554811	3.863825	9.242403
13.B	-2.945738	1.862146	7.301802
14.H	-5.411446	2.600585	6.959364
15.B	-4.237966	3.786080	8.966282
16.H	-4.886272	4.779788	9.053349
17.B	-4.763035	2.169665	9.458607
18.I	-6.786351	1.825987	10.285600
19.B	-1.967089	2.418807	9.931681
20.H	-0.992845	2.425209	10.609923
21.B	-2.230176	1.114089	8.735552
22.H	-1.402797	0.271257	8.614537
23.H	-0.875589	2.934718	7.920791

Table S-8. The optimized geometry coordinates for $[(C_2B_{10}H_{10})_2]^{2-}$

Atom	X	Y	Z (Angstrom)
1.H	-1.066371	0.898330	9.783460
2.B	-2.330464	1.000451	7.516190
3.H	-1.483791	0.491414	6.864404
4.B	-3.178820	2.426688	6.908300
5.H	-2.901411	2.875310	5.848818
6.B	-3.415400	3.523578	8.297183
7.H	-3.324681	4.697098	8.165066
8.C	-0.699380	3.257664	7.963902
9.C	0.020335	3.050197	6.472550
10.B	-2.067553	1.257260	9.262425

11.C	-2.042916	2.549678	8.179590
12.C	-2.763573	2.756973	9.669872
13.H	3.189317	5.202719	7.097387
14.B	-3.626629	1.322729	10.031736
15.H	-3.711833	0.989866	11.173556
16.B	-3.373326	0.196946	8.690256
17.H	-3.312966	-0.982861	8.855892
18.B	-4.072328	0.939869	7.235643
19.H	-4.519744	0.291881	6.338800
20.B	-4.751594	2.514541	7.702745
21.H	-5.687890	3.010894	7.154802
22.B	-4.459320	2.722441	9.434776
23.H	-5.139610	3.390869	10.150679
24.B	-4.885178	1.122178	8.795980
25.H	-5.932221	0.604533	9.042658
26.B	0.672971	2.284205	7.845457
27.H	0.582801	1.110609	7.977884
28.B	0.437582	3.381049	9.234665
29.H	0.162593	2.932622	10.295069
30.B	-0.411298	4.807018	8.626688
31.H	-1.257356	5.316542	9.279023
32.B	-0.675442	4.549715	6.880564
33.H	-1.676864	4.908869	6.360242
34.B	1.716213	3.084767	6.706795

35.H	2.396039	2.415858	5.990924
36.B	2.009667	3.293112	8.438667
37.H	2.946254	2.796643	8.986100
38.B	1.330841	4.867760	8.905886
39.H	1.778891	5.516160	9.802076
40.B	0.630699	5.610286	7.451662
41.H	0.570137	6.790057	7.285835
42.B	0.882968	4.484303	6.110122
43.H	0.967353	4.817019	4.968222
44.B	2.142492	4.685113	7.344956

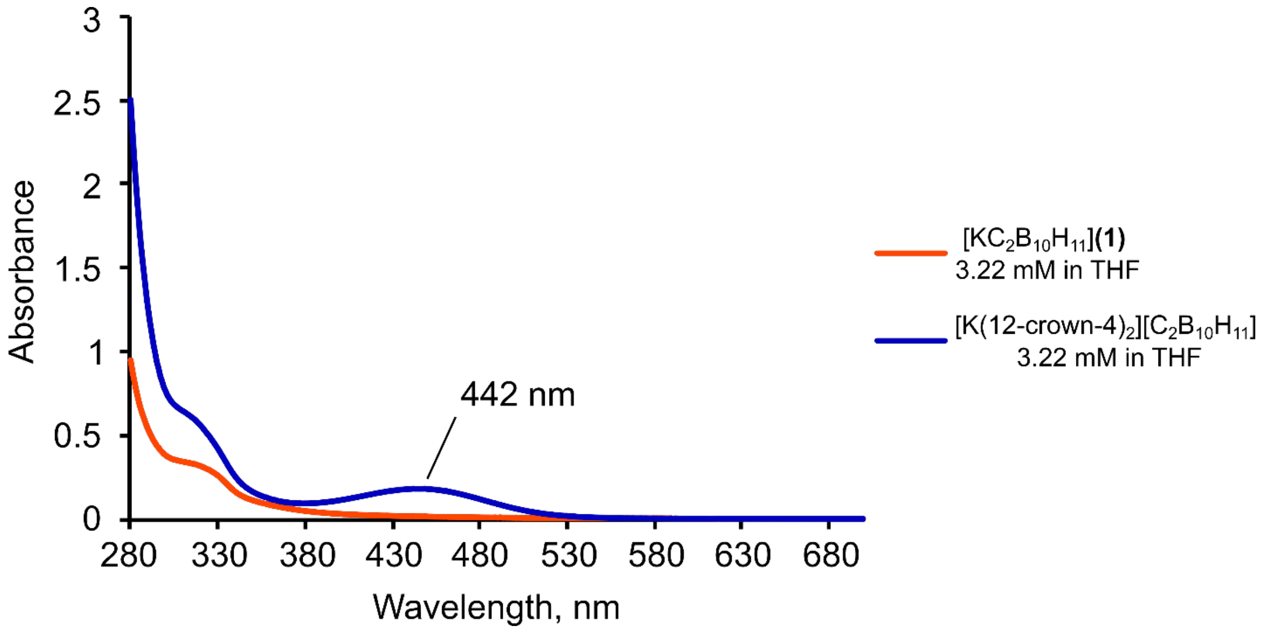

Figure S-24. Absorption spectra of $KC_2B_{10}H_{11}$ (**1,** 3.22 mM in THF) before and after the addition of 2 equiv. of 12-crown-4.

References.

[1] S. Ren, Z. Xie, *Organometallics* **2008**, *27*, 5167–5168.
[2] J. S. Andrews, J. Zayas, M. Jones, *Inorg. Chem.* **1985**, *24*, 3715–3716.
[3] G. te Velde, F. M. Bickelhaupt, E. J. Baerends, C. Fonseca Guerra, S. J. A. van Gisbergen, J. G. Snijders, T. Ziegler, *J. Comput. Chem.* **2001**, *22*, 931–967.
